# Skeletons of swiftly swimming sharks: Three‐dimensional analysis of lamniform vertebral morphology and mineral architecture

**DOI:** 10.1111/joa.70209

**Published:** 2026-07-14

**Authors:** Jamie L. Knaub, Madisan Biordi, Emma Pawlik, Michelle Passerotti, Lisa J. Natanson, Tricia Meredith, Marianne Porter

**Affiliations:** ^1^ Department of Biological Sciences Florida Atlantic University Boca Raton Florida USA; ^2^ Apex Predators Program, Northeast Fisheries Science Center National Oceanic and Atmospheric Administration Narragansett Rhode Island USA; ^3^ FAU Lab Schools, College of Education Florida Atlantic University Boca Raton Florida USA

**Keywords:** cartilage, centra, micro‐computed tomography, mineralized tissue

## Abstract

Order Lamniformes consists of 15 extant shark species that are ecologically diverse and utilize different swimming modes and speeds. Family Lamnidae includes the shortfin mako, porbeagle, and white shark which are fast, athletic sharks that swim using oscillations confined to the caudal body and fin. Other lamniforms, like the common thresher shark (Alopiidae), sand tiger (Carchariidae), and basking shark (Cetorhinidae), swim via oscillations that begin anteriorly, impacting a greater proportion of the axial body. Swimming oscillations subject the body to repeated bending cycles, including the cartilaginous vertebral column, the main longitudinal axis of the body. Vertebrae are mineralized with the amount and arrangement varying among species. We investigated morphological variation in lamniform shark vertebrae to understand adaptations to locomotive demands among species. We examined vertebral morphology and mineral architecture of lamniform centra across three body regions (anterior, middle, and posterior) and among six species (shortfin mako, porbeagle, sand tiger, white, common thresher, and basking shark) through micro‐computed tomography scans. We analyzed morphology and structure of 139 vertebrae from 24 sharks using meristics, principal component analyses, and 3D landmark‐based geometric morphometrics. Through 3D quantification, we identified regional patterns in centrum size and mineral amount which also varied across shark families. In the lamnids, centra morphometrics are largest in the mid‐body and decrease posteriorly simultaneous with increased counts of lamellae. Together, these trends suggest the middle body region is stabilized while allowing for rapid lateral oscillations at the precaudal pit. Cranio‐caudally compressed centra with high quantities of lamellae were characteristic for common thresher shark centra, likely to support loading in multiple planes from extreme axial bending during tail‐whipping behaviors. In the sand tiger and basking shark, we quantified opposing trends—large anterior centra decreased in size along the column with reduced mineralization for slow swimming. We calculated scaling relationships of mineral volume with shark size and identified a negative allometric relationship, suggesting adult sharks may adapt internal architecture rather than contributing to overall centrum size. This comprehensive analysis of calcified structure in lamniform shark centra provides a greater understanding of skeletal tissues and the adaptation of mineralized cartilage to support swimming and ecological needs.

## Graphical abstract

We examined morphology, 3D arrangement, and mineral amount in cartilaginous vertebrae from four lamniform shark families (Alopiidae, Carchariidae, Cetorhinidae, and Lamnidae). We identified family‐specific mineralization trends, and regional patterning of structures along the column. The mineral variation quantified likely reflects the adaptation of cartilaginous centra for locomotion and ecological needs in lamniform sharks, such as breeching out of water and Alopiidae tail‐whipping.

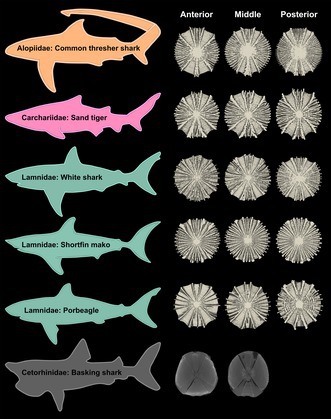

## 1 INTRODUCTION

**FIGURE 1:**
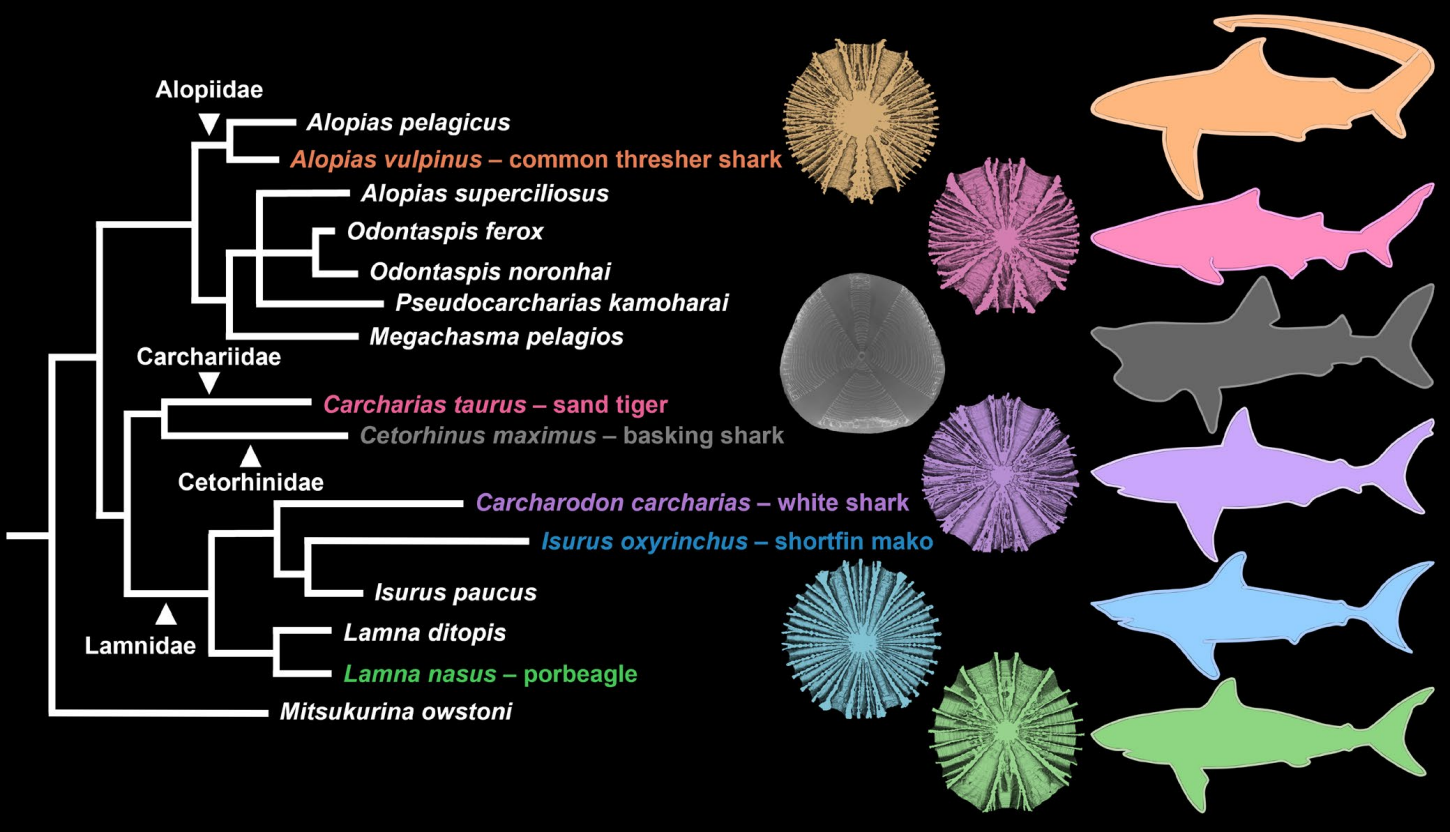
Phylogenetic tree of lamniform sharks. Species examined in this study are listed in color with common names. A transverse model of a centrum was created for each species via the *Segmentation Editor* module in 3D Slicer and is shown next to an outline of the respective species. A transverse section of the micro‐CT scan for the basking shark is shown due to the poor mineralization and difficulty of creating a segmentation. Tree adapted from (Naylor et al., 2012).

Order Lamniformes, the mackerel sharks, is a small yet diverse monophyletic group (Figure 1). It includes family Lamnidae whose members have regional endothermy, the ability to maintain elevated temperature in centralized red muscle and organs (Bernal et al., 2001, 2012; Dickson & Graham, 2004; Ferrón, 2017). The metabolic heat conserved from regional endothermy permits fast contraction of muscle fibers, supporting elevated cruising speeds and high aerobic activity in these sharks (Aalbers et al., 2010; Dickson & Graham, 2004; Gemballa et al., 2006; Saraiva et al., 2023; Watanabe et al., 2015). A distinct characteristic of lamnid species (*Carcharodon*, *Isurus*, and *Lamna*) is the convergence on a tuna‐like body plan and thunniform swimming style, where lateral movement is restricted to the caudal peduncle and tail (Donley et al., 2004; Motani & Shimada, 2023; Sfakiotakis et al., 1999; Webb, 1984). The tapered body morphology and muscular anatomy of lamnids confine swimming‐related loading to the caudal‐most portion of the body.

The thresher sharks, family Alopiidae, include the common thresher shark (*Alopias vulpinus*), which has convergently evolved a medial position of red muscle independent of the lamnid sharks (Bernal et al., 2003; Carey et al., 1985; Donley et al., 2004). Common thresher sharks and other lamniform species, such as the sand tiger (family Carchariidae; *Carcharias taurus*) and the basking shark (family Cetorhinidae; *Cetorhinus maximus*), utilize body oscillations occurring in the posterior half of the body, generally considered the carangiform swimming style (Donley & Shadwick, 2003; Lindsey, 1978; Maia et al., 2012; Sternes & Shimada, 2020). These sharks experience a loading regime that impacts a greater portion of the axial body compared to lamnids whose swimming movement is more localized. While muscular adaptations have been examined in relation to these loading regimes, variation in the 3D anatomy of the axial skeleton is understudied in lamniform sharks.

**FIGURE 2:**
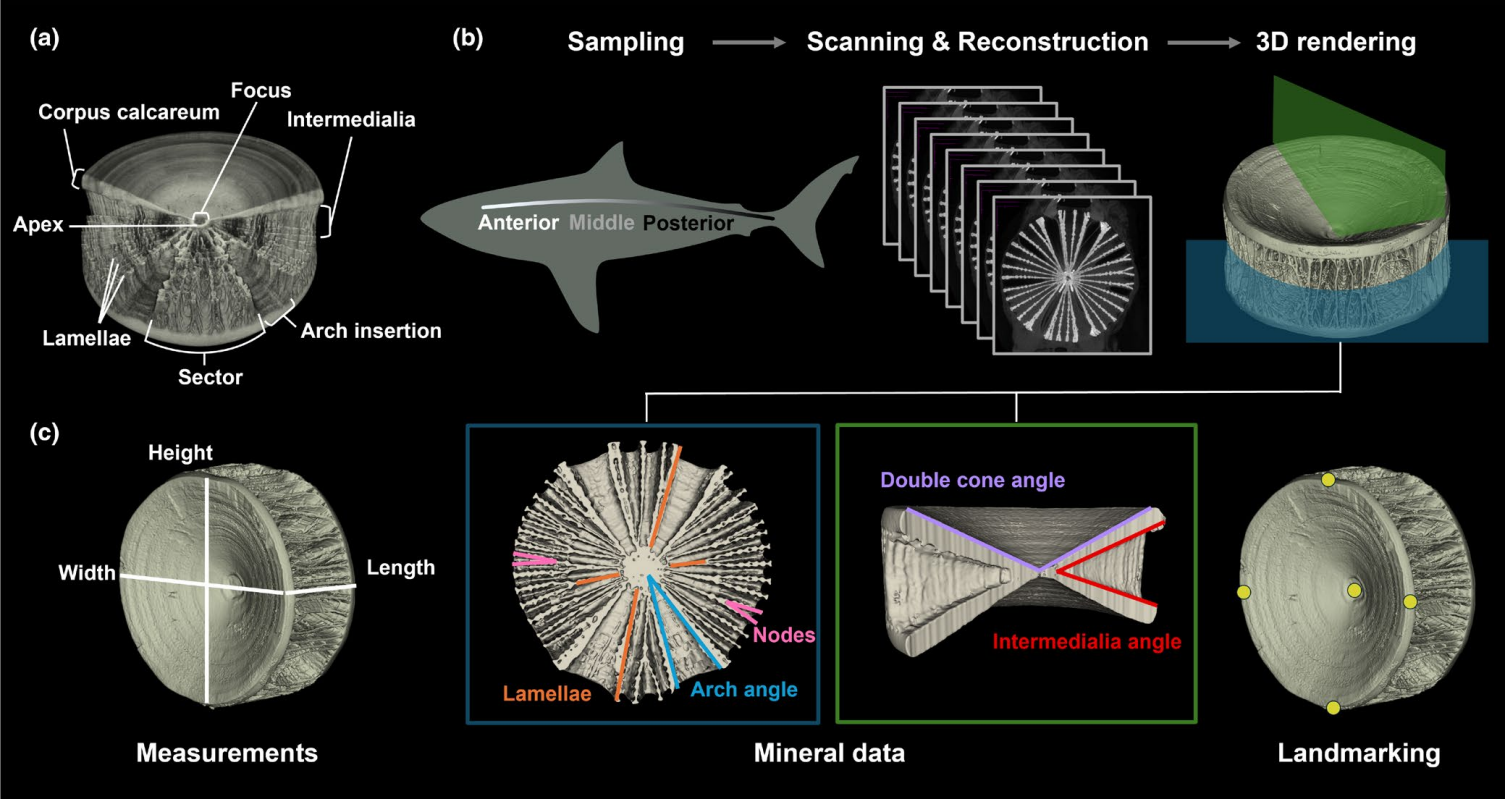
Lamniform shark vertebral morphology and mineral data collection workflow. (a) Three‐quarter view of a lamniform shark centrum comprised of the corpus calcareum (double cone) and intermedialia. (b) Whole body diagram of a lamniform shark depicting approximate sampling locations for anterior, middle, and posterior regions. Centra were micro‐CT scanned and reconstructed image stacks were used to generate 3D renderings of each centrum. (c) Morphometrics collected for each centrum include width, height, and length. Mineral data collected in the transverse plane (blue cross‐section) included lamellae and node counts, and arch angles. Mineral data collected in the mid‐sagittal plane (green cross‐section) included double cone and intermedialia angles. A subset of vertebrae were selected for geometric morphometric analysis via 3D landmarking.

The main body axis of sharks is the cartilaginous vertebral column that experiences millions of bending cycles over a lifetime of swimming (Watanabe et al., 2012). The column is comprised of consecutive cylindrical vertebral centra, separated by intervertebral capsules, which act as a hydrostat in response to loading (Home, 1832; Porter et al., 2014; Schmitz, 1995). A single vertebral centrum is constructed of areolar mineralized cartilage that forms a double cone (hourglass) structure (Figure 2a), the corpus calcareum (Dean & Summers, 2006; Kemp & Westrin, 1979; Maisey et al., 2021; Moss, 1977; Ørvig, 1951; Ridewood, 1921). The intermedialia stretch between the walls of the double cone and form four sectors consisting of mineralized plates (lamellae) that occasionally bifurcate at nodes (Berio et al., 2021; Morse et al., 2022; Natanson et al., 2018; Newbrey et al., 2013; Ridewood, 1921). Unmineralized regions alternate within the radiating lamellae, and between the four sectors are two paired unmineralized gaps, the basidorsal and basiventral insertions, which are continuous with the vertebral arches (Ridewood, 1921). The unmineralized regions between lamellae have significantly lower stiffness (by an order of magnitude) compared to mineralized regions, and the vertebral arches do not bear appreciable compressive loads (Porter & Long Jr., 2010; Raja Somu et al., 2024). While the unmineralized regions may have complex mechanical interactions with mineralized regions, here, we focus our study on the variation in mineralized structures of shark vertebrae.

The amount and arrangement of mineral contribute to the mechanical performance of vertebral centra and vary along the column and across shark species (Dean & Summers, 2006; Dingerkus et al., 1991; Ingle et al., 2018; Knaub et al., 2024; Morse et al., 2022; Natanson et al., 2018; Porter et al., 2006, 2007; Ridewood, 1921; Stock et al., 2024; Summers & Long Jr, 2006). While previous work describes significant relationships between double cone angle and mechanical properties (stiffness and toughness), the substantial morphological variation in the intermedialia warrants further investigation (Ingle et al., 2018). Lamellae vary in thickness, amount, and arrangement within centra (Hansen et al., 2013; Knaub et al., 2024; Kozuch & Fitzgerald, 1989; Morse et al., 2022; Natanson et al., 2018; Ridewood, 1921; White, 1937). Mineral density of the intermedialia and corpus calcareum is comparable in lamniform sharks, suggesting the 3D position and quantity of mineralized structures (lamellae and nodes) may be a strong contributor to the mechanical behavior of centra (Morse et al., 2022).

The goal of this study was to examine the morphology, mineralized structures, and 3D shape in lamniform shark centra to better understand the form‐function relationship of vertebral cartilage and swimming‐related loading. We assessed vertebrae along the body from four lamniform families (Lamnidae: *Carcharodon*, *Isurus*, and *Lamna*, Alopiidae: *Alopias*, Carchariidae: *Carcharias*, and Cetorhinidae: *Cetorhinus*; Figure 1). Using high‐resolution micro‐computed tomography (micro‐CT) scans, we quantified centrum size (height, width, length), internal geometry (arch, intermedialia, and double cone angles), and calcified structures (lamellae and nodes). We predicted that centra would be largest (length, width, and height) in the middle region, as previously reported in lamniform sharks, (Natanson et al., 2018) and that lamellae and nodes would not correlate with centrum size, but rather their quantity would increase in the posterior region of the vertebral column. We used a three‐dimensional landmark‐based geometric morphometric analysis to assess the variations in centrum shape and mineral distribution across the six species. We hypothesized that posterior vertebrae would have a distinct arrangement to withstand a greater degree of swimming‐related loading. We expected the three lamnid species (shortfin mako, porbeagle, and white shark) to have comparable vertebral morphology because of their close phylogenetic relationships and thunniform swimming style. We predicted the common thresher shark, sand tiger, and basking shark would have dissimilar vertebral architecture to the lamnids because they are more distantly related and utilize a carangiform swimming mode. While we classify the six species investigated into one of the four major kinematic swimming modes to facilitate comparison, we realize there is considerable research demonstrating that these classifiers are not always accurate and rather locomotion is better described as a continuum due to the substantial variability within and among species (Di Santo et al., 2021; Long & Nipper, 1996). In this study, we use the designations of “thunniform” or “carangiform” as a general grouping for sharks that utilize different proportions of the vertebral column during oscillatory swimming. Finally, we quantified the mineralized volume relative to the total centrum volume, when modeled as a cylinder, to investigate changes in quantity of mineral structures, centrum size, and mineral amount related to growth. We hypothesized that larger sharks would have a greater quantity of lamellae and nodes, and that centrum volume would scale allometrically with shark length, as previously reported in lamniform sharks (Natanson et al., 2018). We predicted that the mineralized portions of centra (mineralized volume) grow at the same rate as whole centra (centrum volume) and would also scale allometrically to shark length.

## 2 MATERIALS AND METHODS

### 2.1 Specimens and vertebral samples

We obtained vertebrae from six species of the order Lamniformes: basking shark (*C. maximus*; *N* = 1), white shark (*C. carcharias*; *N* = 4), shortfin mako (*I. oxyrinchus*; *N* = 12), common thresher shark (*A. vulpinus*; *N* = 6), sand tiger (*C. taurus*; *N* = 2), and porbeagle (*L. nasus*; *N* = 6) (Table 1). For all sharks, we measured fork length over the body from the tip of the rostrum to the fork of the caudal fin (Natanson et al., 2022). Shark source location and capture or collection methods is summarized in Table S1. Samples from four adult common thresher sharks examined in a previous study were also used in the current study (Knaub et al., 2024).

**TABLE 1:** Vertebral samples examined from six lamniform species across three body regions.

Species	Fork length (FL, cm)	Sex	Length‐at‐maturity (FL, cm)	Body size	Anterior	Middle	Posterior	Total vertebrae	Source
Basking shark (*C. maximus*)	728.3^a^	F	M: 775.0 (TL) F: 701.0	Adult	1	1	0	2	(Ebert & Dando, 2024; Matthews, 1950)
Sand tiger (*C. taurus*)	190.5^b^, ^c^	M	M: 162.5 F: 190.0	Adult	3	3	3	9	(Gilmore et al., 1983)
	221.0^b^	F		Adult	3	3	3	9	
					*6*	*6*	*6*	*18*	
White shark (*C. carcharias*)	322.6^b^	M	M: 315.0^d^ F: 411.3 (TL)	Adult	1	1	1	3	(Márquez‐Farías et al., 2024)
	331.0^b^	M		Adult	1	1	1	3	
	377.0	M		Adult	1	1	0	2	
	380.9^b^	M		Adult	1	1	1	3	
					*4*	*4*	*3*	*11*	
Shortfin mako (*I. oxyrinchus*)	182.8^b^	M	M: 182.0 F: 280.0	Adult	1	1	1	3	(Natanson et al., 2020)
	260.4^b^	M		Adult	1	1	1	3	
	291.5^b^	F		Adult	1	1	1	3	
	292.1^b^	F		Adult	1	1	1	3	
	294.6^b^	F		Adult	1	1	1	3	
					*5*	*5*	*5*	*15*	
Porbeagle (*L. nasus*)	224.4^b^	F	M: 174.0 F: 220.0	Adult	1	1	1	3	(Jensen et al., 2002)
	225.7^b^	F		Adult	1	1	1	3	
	234.0	F		Adult	1	1	0	2	
	241.0^b^	F		Adult	1	1	1	3	
	247.6^b^	F		Adult	1	1	1	3	
	248.0^b^	F		Adult	3	3	3	9	
					*8*	*8*	*7*	*23*	
Common thresher shark (*A. vulpinus*)	194.6^b^	M	M: 188.0 F: 216.0	Adult	0	14	6	20	(Gervelis & Natanson, 2013)
	208.0^e^	M		Adult	0	0	19	19	
	209.9^e^	M		Adult	0	4	0	4	
	226.0^b^	M		Adult	7	4	5	16	
	229.0^b^, ^e^	F		Adult	0	3	3	6	
	241.9^e^	F		Adult	0	5	0	5	
					*7*	*30*	*33*	*70*	
	24 sharks				31	54	54	139 vertebrae	

*Note*: Published length‐at‐maturity data (see source) was used to designate specimens as an adult. All measurements listed are fork lengths (FL; cm) unless otherwise noted. When a length range was provided, we used the median of the range. The anterior, middle, and posterior columns represent the number of vertebrae sampled from the respective body region and are summarized as a total for each species in italics. The total number of vertebrae sampled per individual is listed in the subsequent total vertebrae column and is summarized as a total for the whole study at the bottom of the table. All sharks except the basking shark were used in the mineral architecture principal component analysis (Figure 7).

^a^ Used in landmarking only.

^b^ Landmarked after mineral analysis.

^c^ Estimated FL.

^d^ Calculated FL based on TL.

^e^ From Knaub et al. (2024).

We extracted one to 19 vertebrae from three locations along the column (when feasible): anterior: cranial to the insertion of the first dorsal fin, middle: ventral to the first dorsal fin, and posterior: between the second dorsal fin to the insertion of the caudal fin (Table 1, Figure 1b). Five common thresher sharks had samples taken from under the first dorsal fin and the precaudal pit, which aligns with the middle and posterior body regions. No anterior vertebrae were available. Following extraction, we stored vertebrae in a −20°C freezer.

For the basking shark, white sharks, porbeagles, shortfin makos, and common thresher sharks, we assigned a size class to each specimen by comparing individual FL to published length‐at‐maturity data (Table 1). We considered the shark to be an adult if it had reached a FL greater than the length‐at‐maturity values for the respective species and sex (Table 1). The male sand tiger did not have length data available. We estimated the FL using a total length (TL; cm) to centrum radius (CR; mm) regression for the species and then converted TL to FL (Goldman et al., 2006). Based on these calculations, we concluded that the sand tiger was of adult size.

### 2.2 Micro‐CT scanning

We thawed vertebrae prior to scanning and measured the greatest height, width, and length (mm) of each centrum with digital calipers (Knaub et al., 2024). We stacked centra vertically in a plastic canister stabilized with polyurethane foam or synthetic sponges and cling wrap for micro‐CT scanning. We imaged vertebrae using a Bruker SkyScan 1173 micro‐CT system (Kontich, Belgium) at FAU Lab Schools Marcus Research and Innovation Center Berlin Family Bioimaging Lab (RRID:SCR_023805). We used scan acquisition settings consistent with previous micro‐CT methods for shark vertebrae including flatfield correction, 1‐3x frame averaging, 0.2‐degree rotation step, 1.0 mm aluminum filter, 120 kV (voltage), 60 μA (current), 625–1500 ms exposure, and a voxel size between 15 and 36.0 μm (Knaub et al., 2024). We reconstructed 16‐bit TIFF radiographs with 15–30% beam hardening correction, 2–10 ring artifact correction, and 0.2‐degree angular step using Bruker NRecon software (v1.7.1). The basking shark vertebrae exceeded the chamber size of the Bruker SkyScan; therefore, we scanned them at the University of Florida's Nanoscale Research Facility (RRID:SCR_025135) using a Waygate Technologies Phoenix v|tome|x m 240 system (Baker Hughes Digital Solutions GmbH, Hürth, Germany) and associated datos|x acquisition and reconstruction software (v.2.8.2.20099). We used acquisition settings including a 3‐point detector calibration, detector shift, 3x frame averaging, one frame skip, 2451 rotation steps, 0.5 mm tin filter, 170 kV, 825 μA, 333 ms exposure, and 147 μm voxel size. We reconstructed 32‐bit TIFF radiographs to a “.vol” 3D dataset after applying an ROI‐CT filter and a beam hardening correction of 6.5. We exported TIFF image stacks through Hexagon VG Studio Max software (v2022.1). Reconstructed image stacks and imaging metadata fare hosted on MorphoSource https://doi.org/10.17602/M2/L812881.

To confirm the accuracy of the morphometric measurements, we compared centrum width, height, and length data collected with calipers on thawed samples in relation to measurements collected from the micro‐CT scans. Using a *t*‐test, we found no significant differences between the two methods, suggesting that centra did not shrink substantially during scanning. For the remainder of the manuscript, centra morphometrics refers to the width, height, and length measurements we collected with calipers on thawed samples.

### 2.3 Quantifying mineral architecture

We quantified morphometrics and mineral variables for 137 centra from 23 shark specimens (centra and individuals by species: SFM – 15 centra, 5 sharks, P – 23 centra, 6 sharks, W – 11 centra, 4 sharks, ST – 18 centra, 2 sharks, CT – 70 centra, 6 sharks, Table 1). We standardized centrum morphometrics by dividing the measurement by shark fork length, allowing for comparison across specimens. To quantify structures, we generated 3D renderings of centra in Bruker CTVox software (v3.2) and captured three replicate images through the apex of the corpus calcareum (transverse plane) and three mid‐sagittal images (Figure 2b). Prior to structure counts, we renamed each image with a randomly generated alpha‐numeric code to reduce unintentional bias (Knaub et al., 2024). We selected images at random and counted the number of lamellae and nodes, and measured arch angles, intermedialia angles, and double cones angles (Figure 2c) using ImageJ (Schneider et al., 2012). For counts, each standalone mineralized plate radiating from the apex was considered one lamella, and any point of branching on lamellae was counted as a node. For each centrum, we averaged counts and angle measurements across the three replicates. We excluded the basking shark from these measurements due to the poor calcification within the centra and lack of mineralized structures, which has been previously reported for this species (Ridewood, 1921; Sadowsky, 1973).

### 2.4 Geometric morphometrics

We conducted a 3D geometric morphometric analysis using landmarks to examine the spatial differences in vertebral mineral architecture across lamniform species and body regions (Figure 2c). We landmarked one anterior, middle, and posterior centrum from adult shortfin mako, porbeagle, sand tiger, and white sharks (Table 1). During landmarking, we further examined one white shark anterior centrum and determined to exclude it from the analysis due to abnormal mineralization. We landmarked one anterior, middle, and posterior centrum from one adult common thresher shark, and middle and posterior centra from two additional adult common thresher sharks. We landmarked an anterior and middle centrum from a single basking shark; no posterior vertebrae were available, totaling 53 centra (19 specimens) used in the analysis.

For each centrum, we imported image stacks into SlicerMorph, an extension of 3D Slicer (Rolfe et al., 2021). After importing image stacks, we entered the voxel spacing information (mm) and loaded each scan at full resolution. We used the *Segment Editor* module to create a segmentation by thresholding the density of the mineralized centra. We edited each segmentation on an individual basis after visual inspection in 2D and 3D views. When necessary, we manually edited segmentations with small holes or excess protrusions using *Fill Holes*, *Islands*, *Paint*, *Erase*, and/or *Smoothing* tools. We quantified the volume of the mineralized centrum (mineralized volume, mm^3^) using the *Segmentation Statistics* module, which multiplies the number of voxels in the segment by the voxel dimensions. We then exported the segmentation to a model for landmarking.

**FIGURE 3:**
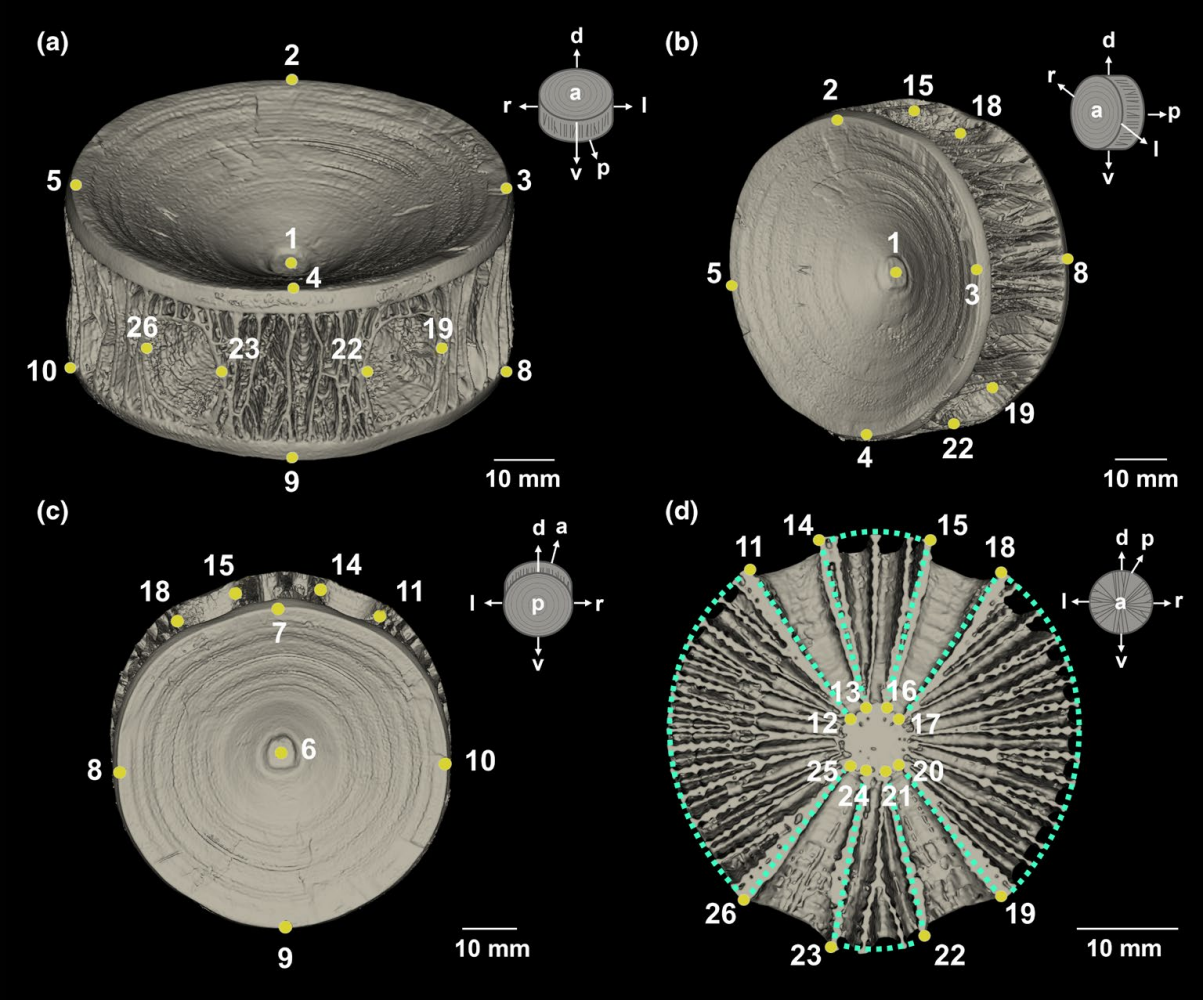
Landmarking placement for geometric morphometric analysis. Landmarks 1–5 were placed on the anterior centrum face, 6–10 on the posterior centrum face, and 11–26 at locations outlining mineral sectors at the double cone apex. Each diagram has an anatomical atlas in the upper right corner for orientation. (a) Three‐quarter ventral view of the anterior face; posterior cone is pointed downwards (not visible). (b) Three‐quarter sagittal view of the anterior face. (c) Caudal view of posterior face. (d) Transverse view at the double cone apex depicting internal landmarks. See footnote in Table 1 for details on specific vertebrae used in the geometric morphometric analysis.

We landmarked each centrum using 26 fixed landmarks in a *Point List* from the *Markups* module in 3D Slicer. We placed landmarks on 2D views and used the 3D viewer to verify landmark location on the model. We placed the first landmark on the cone surface at the focus, or the mid‐point of the double cone structure where the cranial and caudal cones of the corpus calcareum articulate (Figure 3). We placed the next four landmarks (2 through 5) at the distal‐most edge of the double cone in the sagittal and coronal planes. We placed the next five points (6 through 10) in mirrored locations on the opposing cone surface. Using the remaining landmarks (11 through 26), we outlined the exterior edges of the intermedialia and arch insertions on the transverse image that correlated with the double cone apex (Figure 3). We could not landmark all lamellae because of the large variation in quantity between centra, and their position is not homologous across samples (Bookstein et al., 1991; Hallgrimsson et al., 2015; Oxnard & O'Higgins, 2009). However, by landmarking the exterior edges of each intermediale, we can analyze shape changes in the mineral architecture in four sectors (dorsal, ventral, and two lateral; Figure 3d).

### 2.5 Statistical analyses

We analyzed data using R 4.4.2 (R Core Team, 2025). Morphometrics and mineral data variables for all lamniform species did not meet normality and homogeneity assumptions, even after data transformations. We used a ranked one‐way ANOVA to explore morphometrics across body regions (anterior, middle, and posterior) and two‐way ranked ANOVAs to analyze body region, species (shortfin mako, porbeagle, white shark, sand tiger, and common thresher shark), and an interaction of body region * species for mineral counts (lamellae and nodes) and vertebral architecture (arch, double cone, and intermedialia angles). We used Tukey's HSD post hoc tests to identify significant differences between effects. We used OLS regression models to explore the relationships between lamellae, nodes, and centrum morphometrics (all variables log‐transformed). We examined all mineral data variables and centra morphometrics (standardized to shark fork length, see Table S3) using a principal component analysis (PCA; *prcomp* function, scale = True). We used ANOVAs to assess the significance of body region and species on the main PC axes.

To analyze 3D geometric morphometric data, we performed a *Generalized Procrustes Analysis* (GPA) in SlicerMorph and imported the landmarks and GPA output into R using the “SlicerMorphR” package. Using the “geomorph” package, we used Procrustes ANOVAs (*procD.lm* function) and subsequent post hoc tests (*pairwise* function) to assess the significance of swimming mode, body region, and species on 3D shape variation. We used the *gm.prcomp* function to run a PCA and created variance plots using the *scatter3D* function from the “plot3D” package.

To explore mineral structure in relationship to growth, we used OLS regressions on log‐transformed variables, fork length as the body size metric and lamellae count, node count, centrum volume, and mineralized volume as the dependent variables. We followed methods from (Natanson et al., 2018) and calculated centrum volume (*V*_c_, mm^3^), by modeling it as a cylinder as follows:

$$V_{C}=\pi \times \text{centrum length}\;\left(\mathrm{mm}\right)\times {\left(\frac{\text{centrum width}\;\left(\mathrm{mm}\right)}{2}\right)}^{2}$$

We calculated confidence intervals (CIs) for regressions between fork length and volumes (cylindrical and mineralized) using the *confint* function in R and considered a slope allometric if the isometric slope fell outside the confidence interval. Volumes are three‐dimensional whereas fork length is a linear measurement; therefore, an expected isometric slope would be 3 (Natanson et al., 2018; Vogel, 2013). To test the assumption that a greater quantity of lamellae increases the mineral amount within a centrum, we divided the mineral volume (mm^3^, the number of segmented voxels multiplied by voxel size) by the cylindrical volume (to control for size). We termed this variable the proportion of mineral and used a log‐transformed linear regression to explore the relationship between lamellae quantity and mineral amount.

## 3 RESULTS

### 3.1 Centrum morphometrics

**FIGURE 4:**
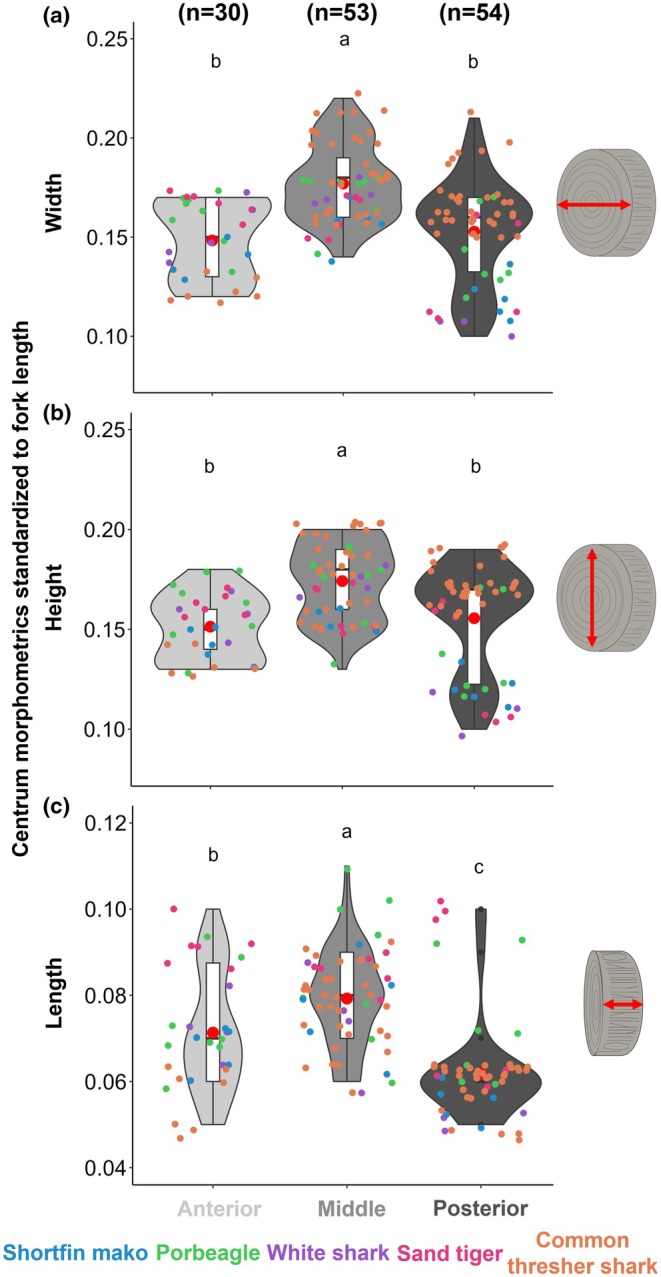
Centrum morphometrics across body regions for 5 lamniform species. Centrum (a) width, (b) height, and (c) length (mm) were standardized by dividing each measurement by shark fork length. Violin colors increase in saturation across body regions. Violin width depicts distribution of points, and a boxplot within each violin indicates quartile ranges. The median and mean are shown as a black horizontal line and red point, respectively. Tukey post hoc results are shown above each violin and indicate significant differences. N values represent the number of centra examined for each region. The number of centra examined for each species is listed in Table 1.

We analyzed 137 centra from 23 individuals of five lamniform species (excluding the basking shark, Table 1). A comprehensive summary of average (± SD) centrum morphometrics and mineral data across species is provided in Tables S2 and S4, respectively. The one‐way ANOVA model was significant for standardized centrum width (*p* < 0.001), height (*p* < 0.001), and length (*p* < 0.001) and body region (Table 2). Post hoc tests showed that centra were significantly larger (width, height, and length) in the middle region compared to anterior and posterior regions (Figure 4). Anterior and posterior centra were comparable in width and height, but posterior centra were significantly cranio‐caudally compressed (shorter centrum length) than anterior centra.

**TABLE 2:** One‐way ranked ANOVA results for centrum morphometrics across body regions.

Variable	*df*	*F*	*p*
Centrum width	2134	22.183	< 0.001
Centrum height	2134	13.427	< 0.001
Centrum length	2134	35.12	< 0.001

The log‐transformed linear regression models for lamellae count showed a significant relationship with standardized centrum width (*p* < 0.001), height (*p* < 0.001), and length (*p* < 0.001) for all sharks. The regression for lamellae count and centrum width (*R* ^2^ = 0.111) and height (*R* ^2^ = 0.158) showed weak positive correlations, while centrum length showed a weak inverse correlation (*R* ^2^ = 0.128; Figure S1). We plotted family‐specific best‐fit lines for each model (Alopiidae—common thresher shark, Carchariidae—sand tiger, and Lamnidae—porbeagle, shortfin mako, and white shark) and observed varying relationships for all morphometric regressions. To further explore these relationships, we extracted family specific model parameters (Table S3). For the centrum width and height family specific models, Alopiidae showed a significant positive relationship (*p* < 0.001). No families had a significant individual relationship with lamellae count and centrum length.

Unlike lamellae counts, the log‐transformed linear regression models for node count and standardized centrum morphometrics across all sharks were not significant (Table S3). Across families, Alopiidae (*p <* 0.001) had a significant inverse relationship with all morphometrics; no other family had significant relationships with centrum morphometrics and lamellae count (Figure S1).

### 3.2 Lamellae and nodes

**FIGURE 5:**
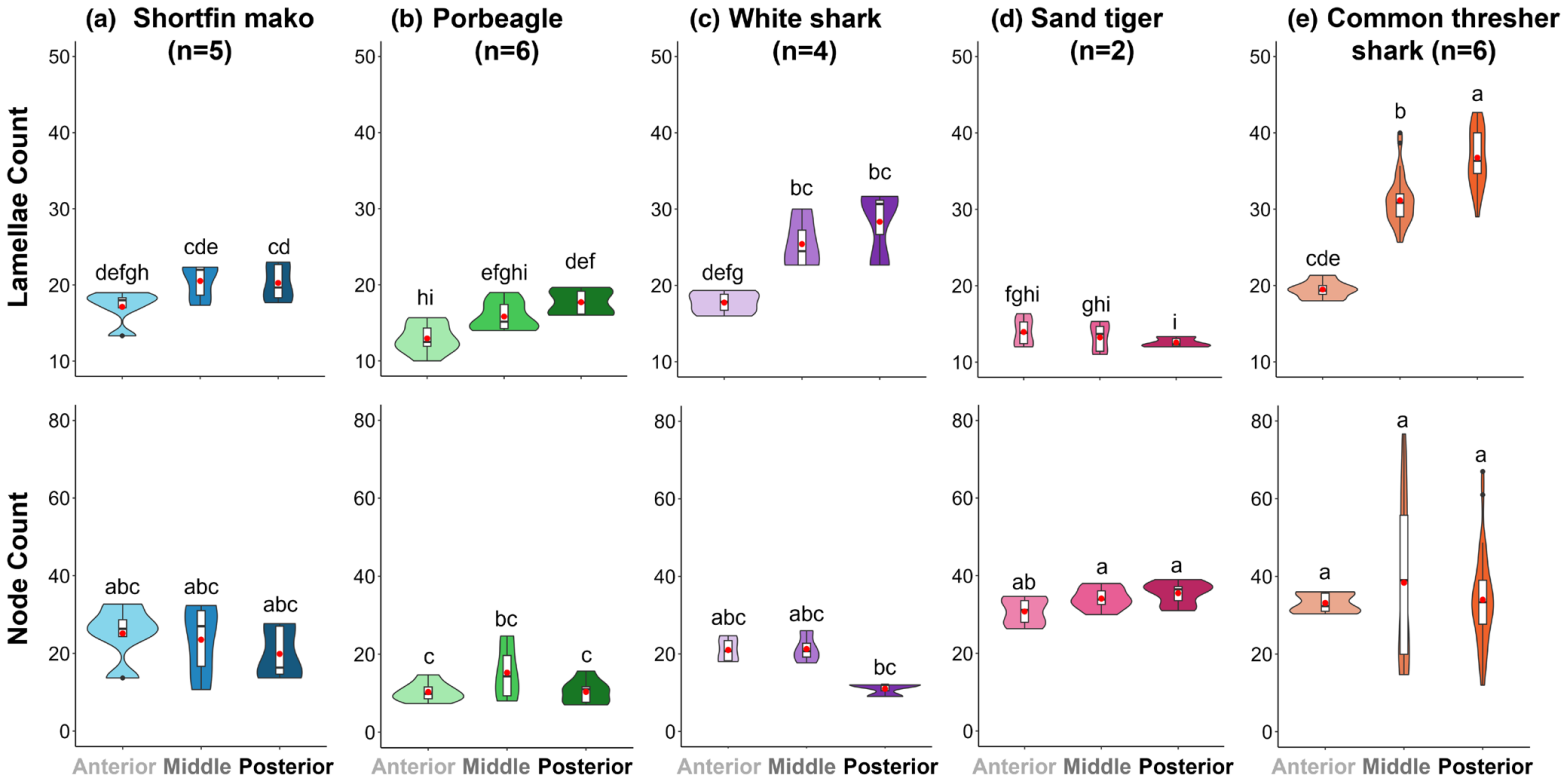
Lamellae and node counts across body regions for 5 lamniform species (a–c: Lamnids, d, e: Non‐lamnids). Violin colors increase in saturation across body regions for each species. Violin width depicts distribution of points, and a boxplot within each violin indicates quartile ranges. The median and mean are shown as a black horizontal line and red point, respectively. Tukey post hoc results are shown above each violin and indicate significant differences. *N* values represent the number of individual sharks examined for each species. The number of vertebrae examined for each region can be found in Table 1.

The two‐way ANOVA model for lamellae count was significant (*p* < 0.001), and the body region effect, species effect, and the interaction term were significant (Table 3, *p* < 0.001). Post hoc tests showed that the common thresher shark had the greatest counts of lamellae across species (average of 29.1 ± 2.7) and within this species, posterior centra had the greatest counts followed by middle and then anterior centra (Figure 5). The white shark had the next highest average lamellae count (23.8 ± 3.3) and showed an increasing number of lamellae when moving caudally down the vertebral column (anterior to posterior). The shortfin mako (average of 19.3 ± 2.4) showed a similar trend with greater lamellae in the middle than the anterior region but had comparable counts of lamellae in the middle and posterior regions. The porbeagle and sand tiger had similar average lamellae counts (15.5 ± 1.8 and 13.2 ± 1.5, respectively) but displayed opposing trends across body regions. The porbeagle had increasing counts of lamellae moving from anterior to posterior regions and the sand tiger had the greatest lamellae counts in the anterior region.

**TABLE 3:** Two‐way ranked ANOVA results for mineral data.

	Lamellae count			Node count			Double cone angle (°)			Intermedialia angle (°)			Dorsal arch angle (°)			Ventral arch angle (°)		
	*df*	*F*	*p*	*df*	*F*	*p*	*df*	*F*	*p*	*df*	*F*	*p*	*df*	*F*	*p*	*df*	*F*	*p*
Whole model	14, 122	82.507	< 0.001	14, 122	9.683	< 0.001	14, 122	28.189	< 0.001	14, 122	71.861	< 0.001	14, 122	25.076	< 0.001	14, 122	28.777	< 0.001
Effects																		
Body region	2	35.291	< 0.001	2	1.172	0.313	2	2.046	0.134	2	10.256	< 0.001	2	18.510	< 0.001	2	16.888	< 0.001
Species	4	135.551	< 0.001	4	29.494	< 0.001	4	47.500	< 0.001	4	140.632	< 0.001	4	29.777	< 0.001	4	56.301	< 0.001
Region * Species	8	8.520	< 0.001	8	0.755	0.643	8	7.660	< 0.001	8	12.164	< 0.001	8	8.010	< 0.001	8	9.794	< 0.001

The two‐way ANOVA model for node count was significant (*p* < 0.001), with species being the only significant effect (Table 3, *p* < 0.001). The common thresher shark had the highest average node count (35.2 ± 10.5) followed by the sand tiger (33.5 ± 3.2, Figure 5). Both species had a comparable number of nodes across all vertebral regions. The shortfin mako had the next highest average node count (22.9 ± 7.8) and like the two other species, had comparable counts across all regions. The white shark had an average node count of 17.8 ± 2.9 and the porbeagle had the lowest average count of all species (12.0 ± 4.1), with both species having comparable counts across anterior, middle, and posterior centra.

**FIGURE 6:**
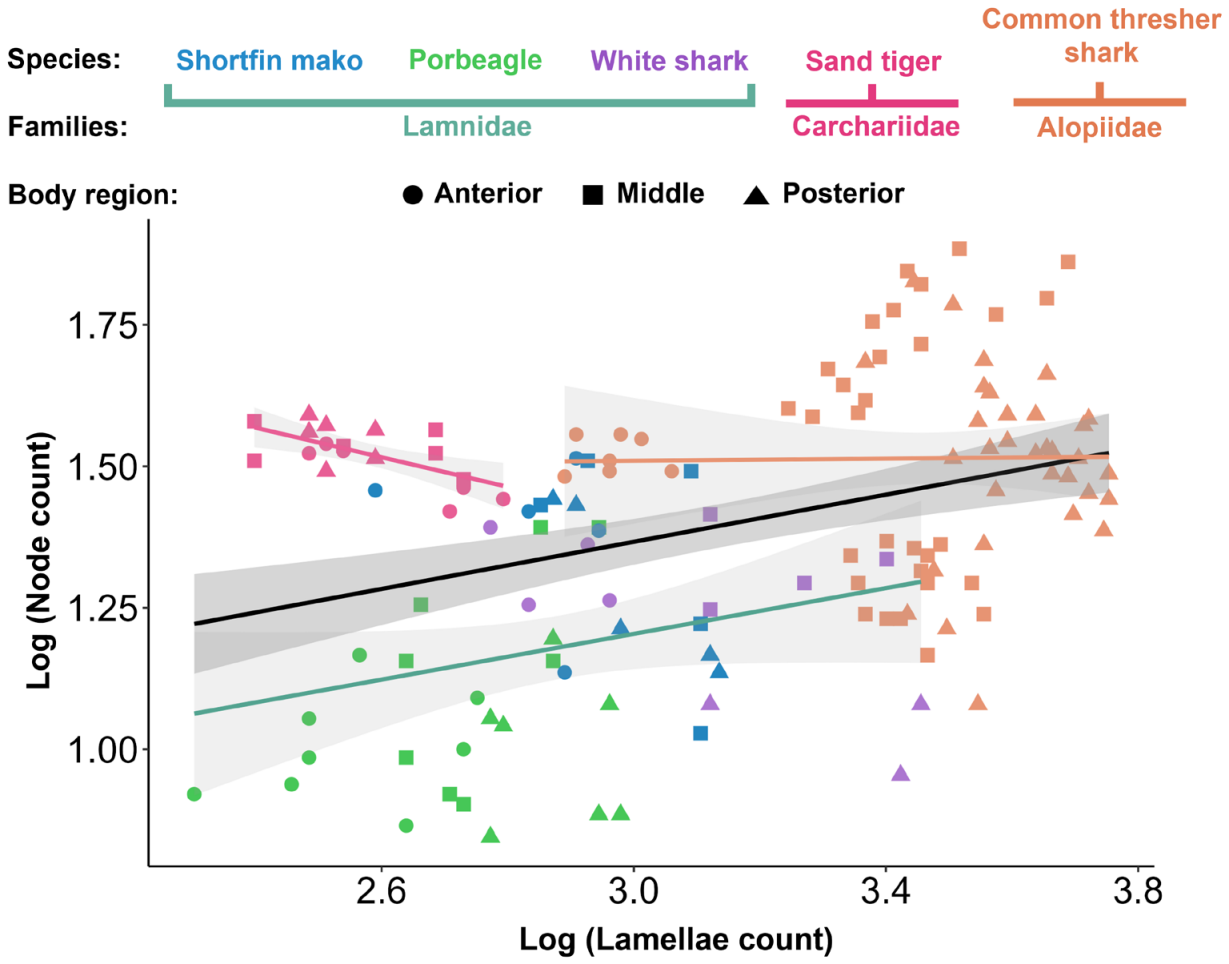
Log‐transformed linear regression of lamellae count and node count. The relationship for all species (black line) was significant (*F*_1,135_ = 18.960; *p* < 0.001). Centra with more lamellae are predicted to have more nodes (*y* = 0.208*x* + 0.742; *R* ^2^ = 0.117). Point shape depicts body region and color represents species. Family specific relationships are depicted by color regression lines (Alopiidae in orange, Carchariidae in pink, and Lamnidae in teal). The shaded gray ribbon for each line represents the 95% confidence interval.

We investigated the relationship between lamellae and node count with a log‐transformed linear regression model and found a weak but significant positive relationship (*R* ^*2*^ = 0.117; *p <* 0.001) for all sharks (Table 4). Family‐specific model metrics showed a significant negative relationship for family Carchariidae (*p* = 0.004); there were no significant relationships for family Alopiidae or Lamnidae (Figure 6). Our regression model exploring lamellae count and the proportion of mineral (mineral volume/cylindrical volume) was significant (*R* ^2^ = 0.059; *p* = 0.003) with a positive relationship for all sharks (Table 4, Figure S2).

**TABLE 4:** Log‐transformed linear regression model results for lamellae quantity in relation to node counts and the proportion of mineral (cylindrical volume/mineral volume).

Lamellae vs	Nodes			Proportion of mineral		
Intercept		0.742			−1.54	
Slope		0.208			0.135	
**All sharks**	***df***	***F***	***p***	***df***	***F***	***p***
	1135	18.960	< 0.001	1135	9.486	0.003
*R* ^*2*^		0.117			0.059	
**By family**	***df***	***F***	***p***	***df***	***F***	***p***
Lamnidae	47	3.127	0.084	47	8.692	0.005
*R* ^*2*^		0.042			0.138	
Carchariidae	16	11.260	0.004	16	10.990	0.004
*R* ^*2*^		0.377			0.370	
Alopiidae	68	0.007	0.933	68	1.026	0.315
*R* ^*2*^		−0.015			0.0004	

### 3.3 Cone and arch angles

**FIGURE 7:**
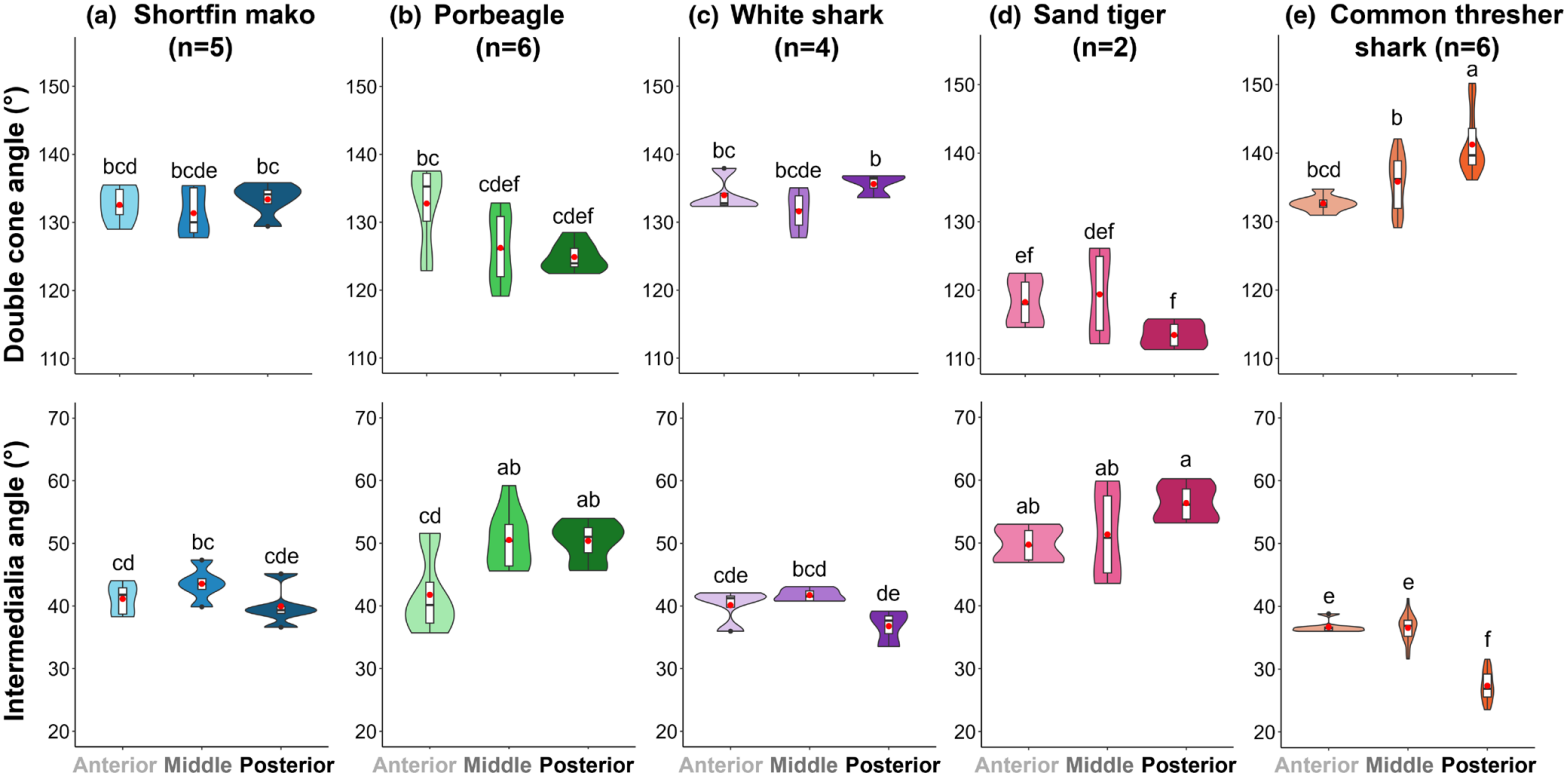
Double cone and intermedialia angles across body regions for 5 lamniform species (a–c: lamnids, d, e: non‐lamnids). Violin colors increase in saturation across body regions for each species. Violin width depicts distribution of points, and a boxplot within each violin indicates quartile ranges. The median and mean are shown as a black horizontal line and red point, respectively. Tukey post hoc results are shown above each violin and indicate significant differences. N values represent the number of individual sharks examined for each species. The number of vertebrae examined for each region can be found in Table 1.

When examining double cone angles, the two‐way ANOVA model was significant (*p* < 0.001) with species (*p* < 0.001) and the interaction term being significant effects (Table 3, *p* < 0.001); body region was not a significant effect. The common thresher shark had the largest average double cone angle (136.6° ± 3.2°), and angles were largest in posterior centra and decreased in middle and anterior centra (Figure 7). The white shark and shortfin mako had comparable averages (133.7° ± 2.6° and 132.4° ± 2.9°, respectively) and displayed a similar trend across body regions; middle centra had smaller double cone angles than anterior and posterior centra. The porbeagle had the next largest angles (128.1° ± 4.4°) which decreased in size moving from anterior to middle centra with the middle and posterior regions having comparable angles. The sand tiger had the smallest average double cone angles (117.0 ± 4.0°) with smaller angles in posterior centra.

The two‐way ANOVA model assessing intermedialia angles was significant (*p* < 0.001) with the body region effect, species effect, and interaction term being significant (Table 3, *p* < 0.001). Post hoc tests showed that the sand tiger had the largest intermedialia angles (average 52.5° ± 4.4°) which were comparable across anterior and middle regions and increased in the posterior region (Figure 7). The porbeagle had the next largest intermedialia angles (average 47.6° ± 4.7°) which were significantly larger in middle and posterior centra compared to anterior centra. The shortfin mako and white shark had comparable averages (41.6° ± 2.8° and 39.6° ± 2.3°, respectively) and similar trends across body regions; middle centra had larger intermedialia angles and posterior centra had smaller intermedialia angles compared to anterior centra. The common thresher shark had the smallest intermedialia angles (33.5° ± 1.8°). Anterior and middle centra were comparable and posterior common thresher shark centra had significantly smaller intermedialia angles.

The two‐way ANOVA model for dorsal arch angle was significant (*p* < 0.001), with the body region effect, species effect, and interaction term also being significant (Table 3, *p* < 0.001). The sand tiger had the greatest average dorsal arch angles of all species (28.1° ± 1.8°, Figure S3). Dorsal arch angles were larger in middle and posterior centra than anterior centra. The porbeagle had the next largest dorsal arches (26.4° ± 3.2°) which decreased in size from anterior to posterior centra. The white shark had the next largest dorsal arch angles (24.0° ± 3.1°) followed by the common thresher shark (22.6° ± 2.0°) and shortfin mako (19.1° ± 3.1°). In both the common thresher shark and shortfin mako, dorsal arch angles decreased across regions from anterior to posterior centra.

Like dorsal arch angles, the two‐way ANOVA model for ventral arch angle was significant (*p* < 0.001) and all effects were significant (Table 3, *p* < 0.001). Sand tiger centra had the largest ventral arches (30.2° ± 1.2°) which were comparable across all body regions (Figure S3). The porbeagle had the next largest ventral arches (28.6° ± 3.2°) which decreased in size from anterior and middle regions to posterior centra. The common thresher shark and white shark had comparable average ventral arches (26.5° ± 1.4° and 23.1° ± 2.9°, respectively). While the white shark has consistent arch angles across all body regions, anterior common thresher shark centra have significantly smaller ventral arches than middle and posterior centra. The shortfin mako had the smallest ventral arch angles (17.5° ± 3.2°) which were larger in the anterior region and comparable across middle and posterior regions.

### 3.4 Mineral data PCA

**FIGURE 8:**
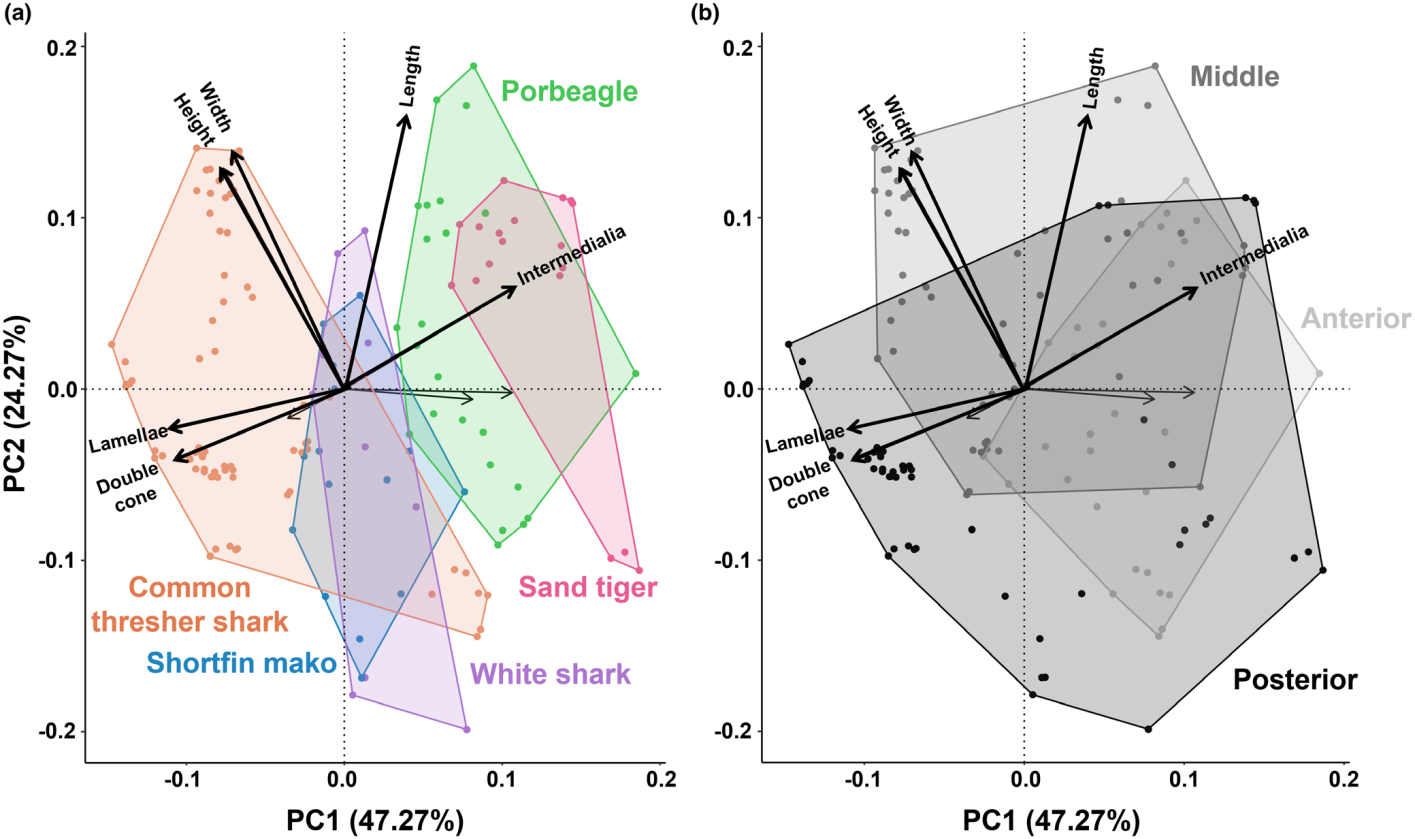
Mineral data quantified for 23 specimens (137 centra) analyzed by PCAs. PC1 accounted for 47.27% variation and PC2 for 24.27% variation (combined 71.54%). Individual points (centra) are framed by group. Main loadings for PC1 were lamellae count, intermedialia angle, and double cone angle. Main loadings for PC2 were centrum morphometrics (height, wight, and length). (a) PCA across lamniform species. We found species significantly different on PCA 1 and 2. Shortfin mako and white sharks had similar mineral characteristics and occupied the middle of the morphospace. Porbeagle and sand tiger centra had similar characteristics to each other but occupied higher PC1 values than all other species. The common thresher shark had some overlap with shortfin mako and white sharks but otherwise occupied the lower PC1 morphospace. (b) PCA across body regions. We found that body region significantly different on PC1 and 2. There was some overlap of all body regions, but middle and posterior centra occupied opposing regions on PC2, while anterior centra fell in between the two.

The mineral architecture variables calculated for 23 sharks in our PCA accounted for a total of 71.54% of the dataset variation across two axes (Table S5, Figure 8). Points were grouped for species and body region to visualize distribution within the morphospace. Main loadings for PC axis 1 (47.27%) were lamellae count, intermedialia angle, and double cone angle (Table S5). PC1 axis was negatively correlated with lamellae count and double cone angle; species with the fewest lamellae and smallest double cone angles (sand tiger and porbeagle sharks) were distributed around greater PC1 values. Intermedialia angle positively correlated with PC1 axis values; the common thresher shark had the smallest intermedialia angles and occupied the negative side of the morphospace. Main loadings for PC2 (24.27%) were centrum morphometric variables (length, width, and height scaled to shark fork length) which all positively correlated with PC2; longer, wider, and taller centra (middle body vertebrae) aligned with greater PC2 values (Table S5). Our ANOVA model showed that body region was significant for PC1 and PC2 (Table 5, *p* < 0.001). Similarly, we found that species was a significant effect for PC1 and PC2 (Table 5, *p* < 0.001).

**TABLE 5:** ANOVA results examining body region and species across mineral data PCA axes 1 and 2.

	PC1			PC2		
	df	*F*	*p*	df	*F*	*p*
*Body region*	2, 134	14.442	< 0.0001	2, 134	32.168	< 0.0001
*Species*	4, 132	80.020	< 0.0001	4, 132	5.805	0.0002

### 3.5 3D geometric morphometric analysis

**FIGURE 9:**
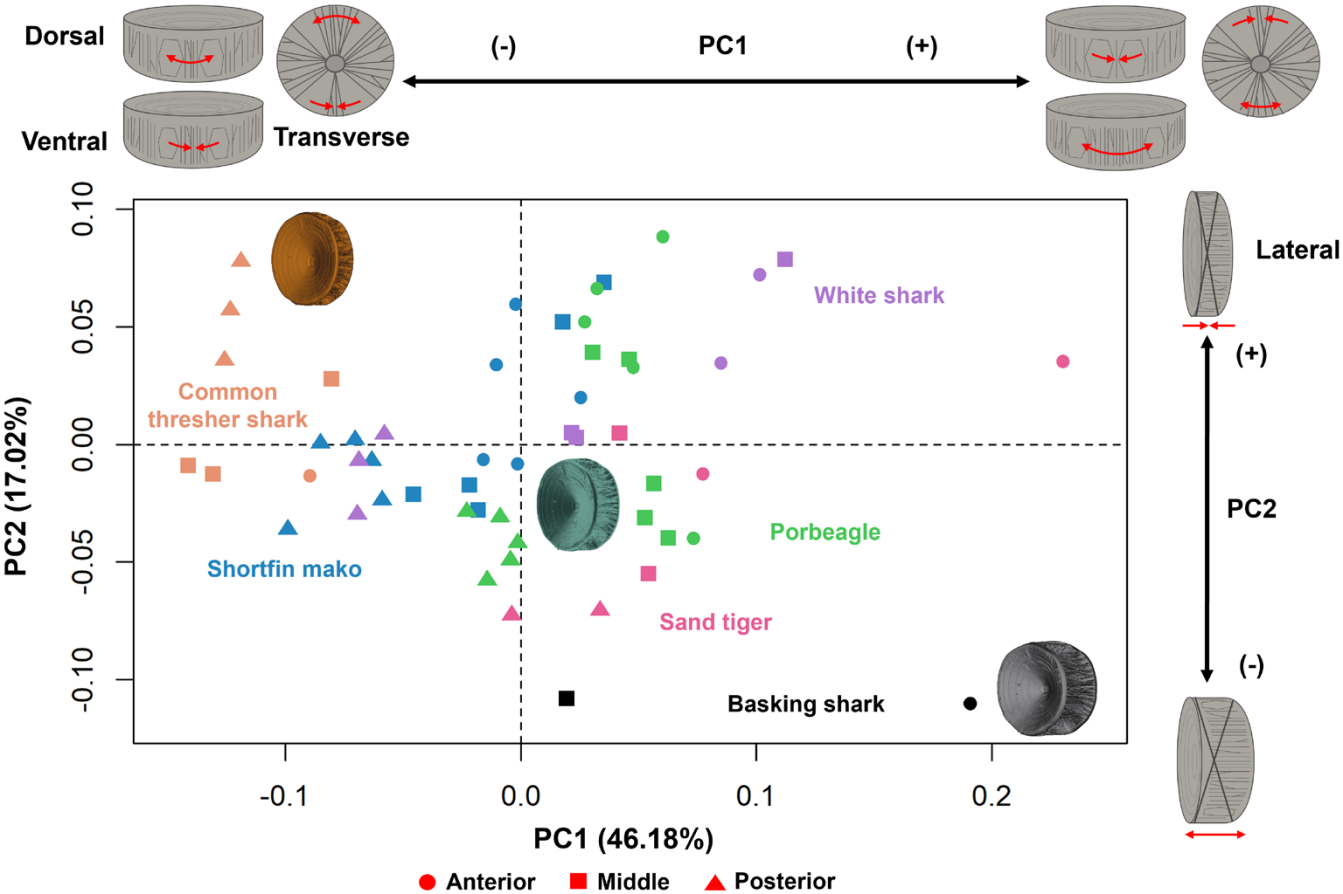
Geometric Morphometric PCA. PC1 explains 46.18% of dataset variation while PC2 covers 17.02%, (63.2% combined). Individual points represent centra (53 from 19 individuals) colored by species. Point shape indicates body region. Variation on PC1 accounted for the size of the dorsal and ventral intermedialia, and PC2 variation represented changes in centrum length and cone concavity. We observed a tight cluster of common thresher shark centra on one end of the morphospace and basking shark centra occupied the opposite end. Meanwhile, substantial overlap of lamnid centra occurred in the center of the morphospace. 3D renderings of centra warped to average landmark locations are shown for the common thresher shark (orange), lamnids (teal), and basking shark (gray). See footnote in Table 1 for details on specific vertebrae used in the geometric morphometric analysis.

We analyzed landmark data for 53 centra from 19 individuals of six lamniform species (Table 1). The variance in landmark placement was examined using 3D plots (Figure S4). The first two principal components of the geometric morphometric PCA collectively explained 63.2% of the dataset variation (Figure 9). The first principal component axis (PC1) accounted to 46.18% of total variance and was driven by landmarks outlining the dorsal and ventral arches. The second principal component axis (PC2) accounted for 17.02% of the total variance and was driven by the distance between landmarks outlining the edge of the cranial and caudal cones. Shape and mineral structure position associated with PC1 and PC2 demonstrated that an increase in PC1 score correlated with shrinking of the dorsal intermediale sector and expansion of the ventral intermediale sector. A decrease in PC2 score correlated with increased distance between the amphicoelous cones and concavity of the cone surfaces; longer centra (basking shark) with more concave cones aligned with lower PC2 scores. The three lamnid species displayed loose clustering with some overlap in the center of the PCA. A clear separation for the common thresher shark and basking shark was observed from other species, with each occupying opposite ends of the morphospace (Figure 9).

The ANOVA for examining the effect of species on 3D morphometric data was significant (Table 6, *p =* 0.001). Pairwise comparisons identified significantly different landmark locations for the common thresher shark relative to all other species (Table 6). The shortfin mako was significantly different from all species except the white shark. The white shark, porbeagle, and sand tiger all shared similar landmarks with each other. The sand tiger was the only species that was not significantly different from the basking shark. The ANOVAs assessing body region (*p =* 0.001) and swimming mode (carangiform and thunniform, *p =* 0.02) were significant, and all pairwise comparisons were significantly different (Table 6).

**TABLE 6:** ANOVA results and pairwise comparisons for geometric morphometric data.

Model type	Model results	Pairwise results	
*Species*	*F*_5,47_ = 8.629, *p* = 0.001	*Z*	*p*
Basking shark – Shortfin mako		2.631	0.002
Basking shark – Porbeagle		2.300	0.014
Basking shark – Sand tiger		1.428	0.088
Basking shark – Common thresher shark		3.325	0.001
Basking shark – White shark		2.427	0.006
Shortfin mako – Porbeagle		2.131	0.016
Shortfin mako – Sand tiger		2.538	0.007
Shortfin mako – Common thresher shark		2.700	0.003
Shortfin mako – White shark		0.773	0.229
Porbeagle – Sand tiger		0.979	0.166
Porbeagle – Common thresher shark		3.589	0.001
Porbeagle – White shark		−0.334	0.626
Sand tiger – Common thresher shark		3.802	0.001
Sand tiger – White shark		1.186	0.128
Common thresher shark – White shark		3.028	0.001
***Body region***	***F***_**2,50**_ **= 6.062, *p* = 0.001**	***Z***	***p***
Anterior – Middle		2.119	0.021
Anterior – Posterior		3.748	0.001
Middle – Posterior		2.604	0.004
***Swimming mode***	***F***_**1,51**_ **= 3.296, *p* = 0.02**	***Z***	***p***
Carangiform ‐ Thunniform		2.020	0.020

### 3.6 Mineral across body size

The log‐transformed linear regression model for lamellae count and fork length was significant (*p* < 0.001; Table S6) with a weak, inverse correlation for all sharks (*R* ^*2*^ = 0.099, Figure S5). We explored family‐specific model parameters and found that Alopiidae (*p* = 0.002) had a significant negative relationship, while Lamnidae (*p* = 0.005) and Carchariidae (*p* = 0.001) had significant positive relationships. In contrast, the log‐transformed linear regression between node count and fork length was not significant (Table S6; Figure S5). When exploring family specific models, we found that family Alopiidae (*p <* 0.001) and Lamnidae (*p* = 0.003) had positive relationships, while family Carchariidae had a significant negative relationship between node count and fork length (*p* = 0.012).

**FIGURE 10:**
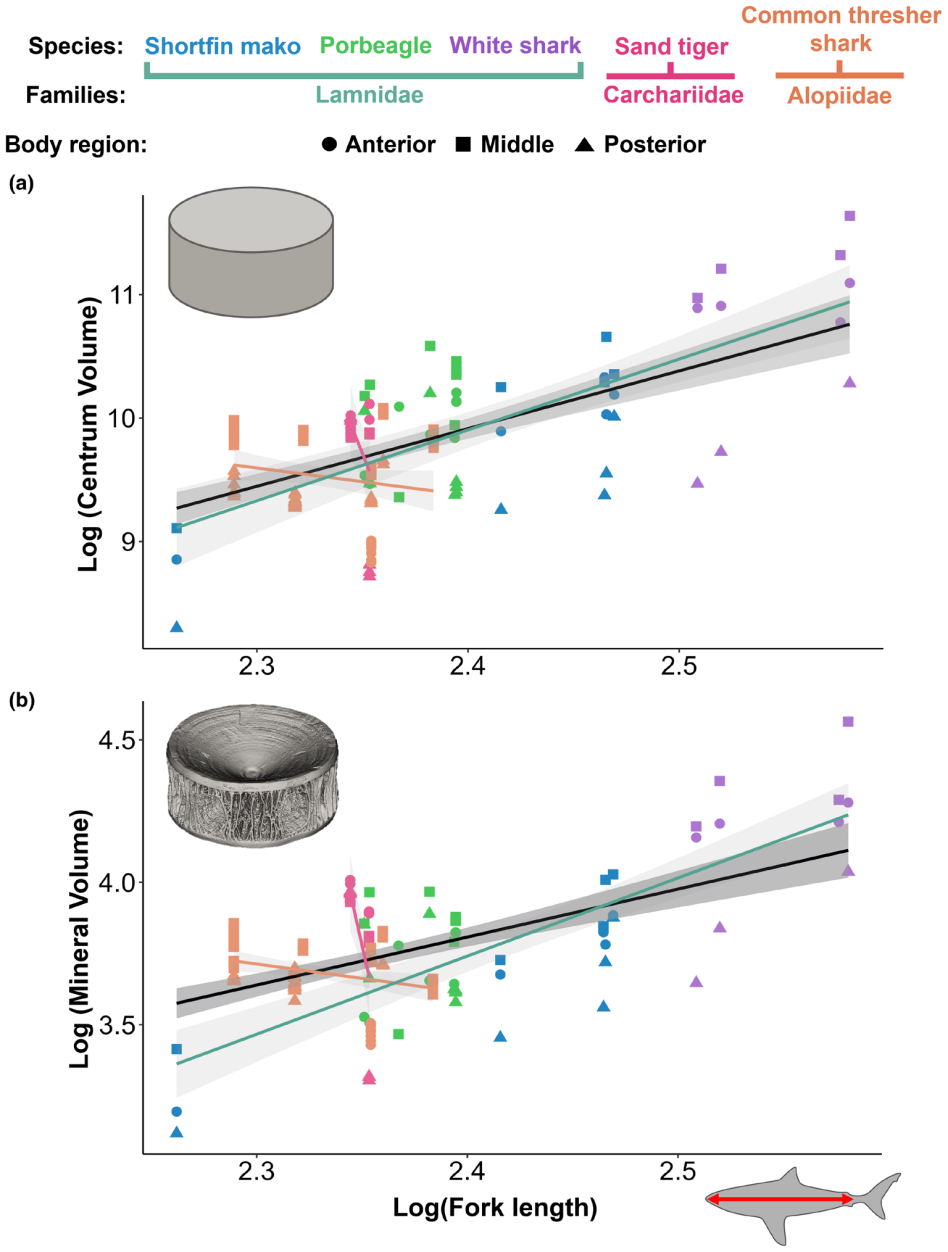
Log‐transformed linear regression of fork length and volumes. Regression models for all species (black line) were significant for (a) centrum volume (*F*_1,135_ = 78.880; *p* < 0.001), and (b) mineral volume (*F*_1,135_ = 61.960; *p* < 0.001). Centrum volume (when modeled as a cylinder) scales with positive allometry (scaling factor of 4.67) in relation to shark length (*y* = 4.672*x –* 1.298; *R* ^2^ = 0.364). In contrast, mineral volume scales with negative allometry (scaling factor of 1.67) in relation to shark length (*y* = 1.684*x –* 0.234; *R* ^2^ = 0.310). Point shape depicts body region and color represents species. Family specific relationships are depicted by color regression lines (Alopiidae in orange, Carchariidae in pink, and Lamnidae in teal). The shaded gray ribbon represents the 95% confidence interval.

The log‐transformed linear regression for centrum volume (modeled as a cylinder) was significant (*p <* 0.001; Table 7). We found that centrum volume scaling with fork length (expected isometric scaling = 3) had a positive allometric relationship with a scaling factor of 4.67 and CIs of 3.63–5.71 (Figure 10). Family Alopiidae and Carchariidae did not have significant family specific relationships, but family Lamnidae had a significant positive correlation (*p <* 0.001) for centrum volume and fork length (Table 7). To better understand the mineralized morphology within centra, we used the volume quantified from segmentations (mineral volume) in a regression with shark fork length. We found that the log‐transformed linear regression model was significant (*p <* 0.001; Table 7). In contrast, the mineral volume and fork length (expected isometric scaling = 3) had a negative allometric relationship, with a scaling factor of 1.68 and CIs of 1.26–2.11 (Table 7). All families had significant individual relationships. Family Lamnidae showed a positive correlation (*p <* 0.001), while family Alopiidae (*p* = 0.008) and Carchariidae (*p* = 0.005) showed negative correlations between mineral volume and fork length (Figure 10).

**TABLE 7:** Volumes and fork length log‐transformed linear regression model results.

Fork length vs	Centrum volume			Mineral volume		
Intercept		−1.300			−0.234	
Slope		4.672			1.684	
**All sharks**	***df***	***F***	***p***	***df***	***F***	***p***
	1135	78.880	< 0.001	1135	61.960	< 0.001
*R* ^*2*^		0.364			0.310	
**By family**	***df***	***F***	***p***	***df***	***F***	***p***
Lamnidae	47	45.760	< 0.001	47	73.420	< 0.001
*R* ^*2*^		0.483			0.601	
Carchariidae	16	3.462	0.081	16	10.620	0.005
*R* ^*2*^		0.127			0.362	
Alopiidae	68	3.014	0.087	68	7.440	0.008
*R* ^*2*^		0.028			0.085	

## 4 DISCUSSION

Our findings support a hypothesis that lamniform shark vertebral morphology and mineral structure reflect differences in swimming mode and ecological needs through regional variation and family‐specific patterning. Fast thunniform swimmers (family Lamnidae) have large abdominal centra that decrease in size and contain more lamellae posteriorly. Fewer nodes in these species may also decrease stress distribution and allow more efficient energy transfer along the column for high‐speed swimming. In contrast, the sand tiger (family Carchariidae) has large anterior centra, fewer lamellae, and high node counts, consistent with a more flexible vertebral column suited to a more maneuverable axial body, likely for slow cruising through convoluted environments. The common thresher shark (family Alopiidae) had high counts of both lamellae and nodes, and cranio‐caudally compressed centra with a high surface area. This morphology likely supports axial bending of the vertebral column in multiple axes for carangiform swimming and both overhead and sideways tail‐whips. Finally, the basking shark (family Cetorhinidae) vertebrae have dramatically reduced mineralization and few, elongated, highly concave centra. The long centrum body and deep cone surface are likely to support slow swimming with high drag forces from feeding at the surface. The arrangement of the mineralized structures and overall centrum shape varied significantly with swimming mode and even at the species level. Overall, our results demonstrate that areolar mineralization (including centrum size, shape, and organization) varies across locomotive strategies in sharks, and broadens our understanding of the form‐functional relationship in calcified cartilage.

### 4.1 Lamnids (family Lamnidae)

Lamnid vertebral size increases with somatic growth; when longitudinal growth slows or stops, sharks will continue to grow in girth and centrum size is proportional to body girth along the column (Natanson et al., 2018). We quantified centra morphometrics across body regions which demonstrate this relationship; the largest centra (width, height, and length) were from the middle region, which aligns with the abdominal cavity, while anterior and posterior centra were significantly smaller (Table S2, Figure 4). Large abdominal centra paired with smaller anterior and posterior vertebrae are mechanically beneficial for the thunniform swimming style for two reasons. Simple beam theory suggests that taller and wider centra in the middle body region provide stability, while a decrease in height and width of posterior centra creates a flexible region at the precaudal pit (Motani & Shimada, 2023). Secondly, significantly shortening posterior centra allows for a greater number of centra and intervertebral capsules in a condensed region, enabling greater flexure (Brainerd & Patek, 1998; Buchholtz, 2001; Motani & Shimada, 2023). Together, the stabilized middle centra and flexible precaudal pit centra align with the generalized body shape and mass distribution of thunniform swimmers to support the significant lateral movements, which are restricted to the posterior region (Sfakiotakis et al., 1999; Webb, 1984).

For family Lamnidae, we did not find significant relationships between centrum morphometrics and lamellae and node counts, suggesting that mineralized structures are unrelated to centrum width, height, and length (Table S3, Figure S1). Next, we interpret trends in the context of families with significant relationships and further discuss mineral structure quantity and centrum morphometrics with family Alopiidae.

The overall quantity of lamellae in the white shark, porbeagle, and shortfin mako followed a regional pattern, where lamellae increased from anterior to posterior regions by approximately 60%, 37%, and 18% for each species, respectively (Figure 5a–c). As hypothesized above, the rapid shortening of centrum length in the posterior region produces a greater range of motion at the precaudal pit. The proportionally smaller vertebral bodies may withstand swimming‐related strain via more lamellae. We found a significant, positive relationship between lamellae count and the proportion of mineral, suggesting centra with more structures have more mineral (Table 4, Figure S2). Mineral amount is known to correlate with material properties in shark centra, and in lamnids specifically, posterior centra are more stiff than anterior centra (Ingle et al., 2018; Porter et al., 2006, 2007). Even when isolated regionally within centra, recent nano‐indentation research observed greater elastic modulus (stiffness) in the posterior intermedialia of shortfin mako when compared to anterior regions (Raja Somu et al., 2024). Stiffer skeletal elements increase the efficiency of energy transfer, and increasing stiffness in a biomimetic vertebral column enabled swimming at higher speeds (Donley et al., 2004; Gemballa et al., 2006; Long et al., 2011; Long & Nipper, 1996; Mchenry et al., 1995; Porter et al., 2007; Webb, 1975). An increase in lamellae quantity—and ultimately stiffness—in lamnid centra would support the fast thunniform swimming style by quickly transferring forces from the musculature to the tail.

The nodes quantified in lamnid sharks were consistent along the vertebral column, but averages varied among species (Figure 5a–c). Shortfin mako and white shark centra had comparable node counts, and the porbeagle had significantly fewer nodes. Similarly, interspecific patterns were quantified with the quantity of growth bands in lamnid centra (Natanson et al., 2018). The circumferential growth bands are visible on the cone surface and throughout the intermedialia and have been discussed in several micro‐CT studies, but whether nodes structurally integrate with growth bands remains unclear (Geraghty et al., 2012; Knaub et al., 2024; Morse et al., 2022; Natanson et al., 2018; Stock et al., 2021, 2022, 2024). Growth band deposition is related to girth, and while we did not find a relationship between nodes and centrum morphometrics, the interspecific relationship between node counts (our study) and banding (Natanson et al., 2018) could suggest a complex or indirect relationship. Higher resolution 3D imaging (possibly nano‐CT) to examine the growth band and node intersection would further explore this relationship. Additionally, our study is limited to the number of nodes at and surrounding the apex of the centrum, and future studies could comprehensively quantify the 3D network of nodes, further untangling relationships between mineral structure and shark growth.

The relationship between lamellae and node count was significant for all sharks, suggesting that centra with more lamellae would bifurcate more (i.e., greater quantity of nodes; Table 4, Figure 6). At the family level, distinct clusters in the regression suggest a trade‐off between lamellae and node quantity. In family Lamnidae, the number of lamellae increased with node count, and the distribution of data shows substantial overlap between the shortfin mako and white shark. Porbeagle centra had little overlap with the other lamnids and had few lamellae and even fewer nodes. This finding may reflect the functional or phylogenetic relationship within family Lamnidae (Figure 1); the shortfin mako and white shark are more closely related (Naylor et al., 2012). In addition to integrating with band pairs, nodes may provide structural support by stabilizing lamellae, like struts of a bridge. Functionally, this could be similar to tesserae, a hypothesized key mechanical component in tessellated elasmobranch cartilage (Fratzl et al., 2016; Jayasankar et al., 2017, 2020; Liu et al., 2014, 2010; Natanson et al., 2018; Seidel et al., 2019, 2020, 2021). For instance, stingray jaws have mineralized struts in areas adjacent to the tooth plates to distribute bite forces (Summers et al., 1998). We hypothesize that the nodes perform a similar function and distribute strain across the centrum. If node formation is a response to mechanical cues, then mineralization will reorient for structural support (Morse et al., 2022). If true, node and lamellae quantity may not be directly correlated due to other structural factors that impact strain within centra. While our node assessment was focused at the double cone apex, quantifying the number of nodes per intermediale sector, the frequency of mineral re‐merging following a node, and the average number of nodes per individual lamella were beyond the foundational scope of this paper (Figure 2). Further investigation on the complexities of the 3D mineralization is warranted.

Double cone angles were similar across body regions in shortfin mako and white sharks, suggesting the cone surface, and possibly the intervertebral capsule encapsulated between cones, is consistent along the body in these two species (Figure 7a,c). Porbeagle centra had comparable double cone angles in the middle and posterior body that were smaller than the double cone angles in anterior centra and the other lamnid species (Figure 7b). These results were unexpected; we had expected a similar double cone morphology to support this thunniform swimming mode utilized by all three species. Lamnid centra with smaller double cone angles have increased stiffness and toughness, possibly due to the increased distance between opposite cones of consecutive centra (Ingle et al., 2018). Porbeagles also have the lowest precaudal vertebral counts of the lamnid sharks we examined; counts averaged by species: porbeagle—87, white shark—105, and shortfin mako—111 (Springer & Garrick, 1964). The porbeagle's shorter vertebral column may compensate for the reduced number of centra and adorning mineralized structures by having smaller double cone angles.

Across body regions, intermedialia angles were largest in the middle region and smallest in the posterior region for the white shark and shortfin mako (Figure 7a,c). Decreasing intermedialia angles would shorten the distance between the highly mineralized amphicoelous cones of the corpus calcareum and may be a morphological constraint of centrum length. This observation is further supported by our findings for the porbeagle, where posterior centra are the longest among the three lamnid species (when standardized to shark length) and had large intermedialia angles (Table S2, Figure 7b). Similar to double cone angles, porbeagle posterior centra have disparate intermedialia angles to potentially offset the mechanics of a shorter vertebral column, while the shortfin mako and white shark have comparable cone morphology and vertebral counts.

The porbeagle had larger average dorsal and ventral arch angles, followed by the white shark; the shortfin mako had the smallest average arch angles (Figure S3a‐c). Previous work on shark caudal fin morphology observed rectangular neural spines and expanded hemal spines exclusively in the porbeagle; however, it was the only lamnid closely investigated (Crofts et al., 2019). The neural and hemal spines are continuous with the arches and insert into centra as unmineralized basidorsal and basiventral cartilage, between intermedialia sectors (Applegate, 1967; Ridewood, 1921; Welton & Farish, 1993). The arch morphology observed in our study may indicate porbeagle centra have larger areas for arch insertion to provide reinforcement for their specific neural and hemal spine morphology.

In all lamnids, arch angle size decreased moving caudally along the column (Figure S3a–c). The smaller arch angles indicate that the unmineralized basidorsal and basiventral insertions are shrinking, and the mineralized intermedialia sectors are expanding. Together, the reduction in unmineralized regions and additional lamellae in posterior centra increases mineral amount and likely centrum stiffness and toughness. Previous mechanical testing studies found a strong positive relationship between stiffness and toughness in shark centra, and vertebral segments tested under high frequencies behave as non‐linear viscoelastic springs (Ingle et al., 2018; Porter et al., 2016). If true in lamnids, posterior centra should absorb more energy during plastic deformation and contribute to greater elastic return, benefiting fast‐swimming species where the lateral movements are confined to the caudal region.

The mineral variable PCA showed that lamellae count, intermedialia angle, and double cone angle drove variation for PC1, while standardized centrum morphometrics had the greatest influence on PC2 (Table S5, Figure 8). The lamnids occupied the middle of the PC1 morphospace, with substantial overlap between the shortfin mako and white shark. The overlap of these two species within the morphospace suggests they share a comparable mineral structure (PC1) and centrum geometry (PC2), presumably due to their close phylogenetic relationship and/or similar ecologies (Compagno, 1984; Naylor et al., 2012). In the morphospace, the porbeagle was located positively for both PC1 and PC2. Relative to the other lamnids, porbeagle posterior centra were longer had a varied shape of the corpora calcera (double cone and intermedialia angles). Both double cone angle and centrum length impact vertebral mechanical behavior (Ingle et al., 2018; Long et al., 2011), and the observed differences in the porbeagle suggest a morphology to support thunniform swimming with fewer vertebrae building the column.

Based on the geometric morphometric analysis, the greatest shape variation across lamniform centra occurs in the dorsal and ventral intermedialia sectors and vertebral length (Table 6, Figure S4, Figure 9). Distinct groupings of sharks occurred throughout the morphospace, and the interspecific distribution between the lamnids was comparable to the mineral data PCA. The most overlap occurred between the shortfin mako and white shark, and the porbeagle aligned with positive PC1 and PC2 space. Additionally, the lamnids occupied a centralized cluster between the other families for both PCAs, suggesting a strong tie between centra mineralization (amount and arrangement) and shape across lamniform sharks.

A surprising result from the geometric morphometric analysis is the redistribution of intermedialia sectors in relation to arch angles (Figure 9). In the lamnids, smaller arch angles moved caudally across body regions, and as arch insertions decrease in size, it would be logical to assume the two lateral intermedialia sectors are expanding to support swimming loads in the posterior region. Side‐to‐side oscillations occurring at the peduncle and caudal fin load the vertebral column in alternating cycles of tension and compression, primarily in the lateral direction. In the posterior region, as the gaps between mineralized sectors shrink (arch angles), the ventral intermediale sector also contracts and the dorsal intermediale sector expands (Figure 9). While this was unexpected, perhaps the larger mineralized area in the dorsal portion of centra supports the anatomy of the caudal fin. The vertebral column and musculature continue from the precaudal pit into the dorsal lobe of the caudal fin, which are stiffer in lamnids than other species (Crofts et al., 2019). The leading edge of the caudal fin dorsal lobe is reinforced with ceratotrichia and in lamnids, a layer of adipose tissue maintains the cross‐sectional hydrofoil shape of the lobe (Lingham‐Soliar, 2005). The expanded dorsal intermediale in posterior centra is oriented with the leading edge of the caudal fin, providing support to the specialized anatomy and streamlined shape of the lunate tail.

For all lamniformes, we found a significant inverse relationship between fork length and lamellae count, but no relationship to nodes, suggesting larger sharks have centra with fewer lamellae but nodes are unrelated to body size (Table S6, Figure S5). We found that all families had individual significant relationships for lamellae and node quantities in relation to fork length, and we interpret family specific trends in greater detail. Family Lamnidae had positive correlations for fork length and lamellae and nodes, such that larger sharks have centra with more mineralized plates that bifurcate more. Both lamellae and node quantity were unrelated to centrum morphometrics in lamnids, suggesting these mineralized structures may provide support primarily for longitudinal growth. Increasing the length of the shark will impact swimming mechanics, and centra may experience greater loading from propelling a larger body. As a result, centra develop more lamellae to support loading, and if node formation is associated with mechanical cues, we hypothesize that the increased loads would also drive lamellae to bifurcate.

A caveat to consider—one would expect a positive relationship between lamellae and nodes with centrum length if these structures correlate with fork length. We did not observe such relationships when considering only the centra, and a different trend may emerge if we were to examine the intervertebral joint length or vertebral counts relative to fork length. The regional morphology of intervertebral joints along the column is unclear. Between two carcharhiniform shark species, intervertebral joint lengths were significantly different but position along the column was not significant, as it only varied in one of the species (Long et al., 2011). However, the same study identified that centrum shape and intervertebral joint length impact vertebral column mechanics when tested in a biomimetic model, suggesting that both variables are crucial. Future work examining regional variation in intervertebral joint length in conjunction to precaudal vertebral counts would further explore this relationship (Springer & Garrick, 1964). In family Lamnidae, the white shark reaches a larger body size and has more lamellae than the other two lamnids, yet it has fewer vertebrae on average compared to the shortfin mako. One would assume that to compensate for this difference, white shark centra are proportionally longer, but our standardized centrum morphometrics showed that shortfin mako and white shark centra are comparable in size across body regions (Table S2). Likely, the intervertebral joints are a crucial component impacting the morphology and mechanics of the vertebral column (Porter et al., 2009, 2014).

We identified a positive allometric relationship between centrum volume and fork length, scaling by a factor of 4.67 (Table 7, Figure 10a). Our scaling factor was slightly higher than what was reported previously (3.41 from Natanson et al., 2018), which is likely attributed to differences in sampling. Our study examined adult sharks of Order Lamniformes, whereas Natanson et al. explored a larger range of body sizes and included species from Order Carcharhiniformes and Squatiniformes. The latter study provides insight over an ontogenetic range (small to large sharks from each species), giving context to the speed of centrum growth across development. Our data suggest that centra grow even faster than body length in the adult phase, possibly contributing more mineral to centrum height and width to support increasing girth while simultaneously experiencing slowed or ceased longitudinal growth. This would especially hold true for lamnid sharks considering they can achieve substantial girths, and we found a significant family specific correlation for Lamnidae.

To improve our understanding of scaling between centra and shark size, we investigated relationships specific to mineral volume. The cylindrical model is solid throughout, while the mineral volume captures a more accurate representation of lamniform centra morphology by including the size of lamellae, and accounting for gaps between lamellae and the concave cone surfaces. We found a significant correlation between fork length and mineral volume for all sharks and identified a negative allometric relationship with a factor of 1.68 (Table 7, Figure 10b). This finding suggests that centra grow slower than longitudinal growth, and the discrepancy in allometric growth between our two volume models is intriguing from a morphological perspective. One interpretation of this finding could simply be the inverse of the positive allometry of the cylindrical volume model; data in this study are limited to adult sharks and centra may be slow to increase in size despite the increasing body length. A second interpretation is that mineral is contributing more to architecture within centra to support a larger body, therefore, the centra are proportionally smaller but have more adorning structures for resist bending forces. For instance, rather than mineral depositing to grow centra dimensions in parallel with shark length, additional structures (e.g., lamellae and nodes) are formed within the intermedialia, resulting in a very small proportional increase in volume relative to fork length.

### 4.2 Sand tiger (family Carcharidae)

Anterior centra were largest (width, height, and length) in the sand tiger and decreased in size along the column with posterior centra being the smallest (Table S2, Figure 4). Centrum morphometrics were comparable across sand tiger specimens in anterior and middle regions, but we observed substantial variation in posterior centra morphometrics between the two individuals. Our study was limited in sample size; we examined three centra per region from two sand tiger sharks. One specimen (221 cm FL) was collected after stranding deceased and the other (190.5 cm FL) was sampled following its lifespan in a managed care facility (Table S1). Despite the difference in fork length between specimens, it is interesting that the anterior and middle centra were of comparable size and only the posterior centra differed in morphometrics. The estimated fork length for the male sand tiger may have introduced error. Captive sand tiger sharks are known to suffer spinal deformities which may vary in association with collection method, swimming patterns, and nutrition (Anderson et al., 2012; Huber et al., 2013; Preziosi et al., 2006; Tate et al., 2013). It is unclear if sand tiger data in this study reflects intraspecific variation or if one specimen is an outlier. Future morphological studies with increased sample sizes of sand tigers are warranted.

Family Carchariidae did not have a significant relationship between centrum morphometrics and lamellae or node counts, suggesting mineral structures are unrelated to centrum size (Table S3, Figure S1). The sand tiger centra had the fewest lamellae of all species examined and we observed comparably low counts between specimens (Figure 5d). Few lamellae would lower mineral amount and stiffness, which has been previously documented in sand tigers (Huber et al., 2013; Porter et al., 2007). Sand tigers may need less stiff vertebrae and fewer lamellae to meet the needs of their relatively slow‐moving lifestyle as they navigate nearshore environments (Compagno, 1984; Smith et al., 2015). Few lamellae (ranging from approximately six to 12) have been quantified for the smalltooth and bigeye sand tigers (*Odontaspis ferox* and *O. noronhai*), which occupy deeper waters than *C. taurus* but navigate similarly convoluted environments (Compagno, 1984; Hansen et al., 2013; Stone & Shimada, 2019). *C. taurus* was once classified in family Odontaspididae with the other sand tiger species but was separated into family Carchariidae after phylogenetic analyses identified a non‐monophyletic relationship (Stone & Shimada, 2019). However, all three species are categorized as carangiform swimmers and have comparably low lamellae counts despite separate phylogenies, supporting a form‐function relationship between centra morphology and swimming mode.

The decreasing lamellae count along the column in sand tigers was unexpected (Figure 5d). Sand tigers will swallow air to maintain neutral buoyancy, owing to their less active swimming style, and may have a reduced need for highly mineralized vertebrae to support swimming loads (Bass & Ballard, 1972; Smale et al., 2012; Smith et al., 2015). In contrast, anterior centra may have more lamellae to support rapid movements such as lateral flexion and head elevation, which enable sand tigers to ambush agile prey (Moyer, 2021; Moyer et al., 2019).

The high node count in sand tiger centra was surprising (Figure 5d). If nodes distribute stress, it is unclear why additional stress distribution would benefit a slower swimming style. Instead, lamellae may diverge possibly due to spatial constraints (Morse et al., 2022). In sand tiger centra, sparse lamellae may increase nodes with more room between neighboring plates. If true, this would further support Carchariidae's inverse relationship between lamellae and node count (Figure 6).

The sand tiger had the smallest average double cone angles and largest intermedialia angles of all species (Figure 7d). The observed inverse relationship of double cone and intermedialia angles suggests the mineralized cone (corpus calcareum) thickens with increasing radial distance. These thickened outer portions of the cone experience higher strain during lateral bending compared to centralized regions. Considering the small double cones in sand tiger centra, a thicker corpus calcareum may be necessary to constrain the altered shape of the intervertebral capsule.

Sand tigers had the largest average dorsal and ventral arch angles across all species (Figure S3d). The unmineralized basidorsal and basiventral insertions in shark centra have been hypothesized to aid in compliant decoupling and dampen fracture energy of the mineralized sectors (Huber et al., 2013; Morse et al., 2022). Larger arch insertions into centra would allow for greater deformation between mineralized sectors during bending, increasing the range of motion and maneuverability. The resulting increase in vertebral column flexibility would be beneficial to sand tigers which utilize a greater proportion of the axial body to swim through convoluted inshore waters.

Within the mineral data PCA, the sand tiger specimens occupied the positive end of PC1, and all but three centra aligned with positive PC2 values (Figure 8a). These three posterior centra were substantially smaller in all dimensions (width, height, and length) compared to the other sand tiger centra. Relative to other species, the smaller centra occupied similar PC2 values as other posterior centra (Figure 8b) and may represent natural variation. Surprisingly, the sand tiger and porbeagle ellipses overlapped despite utilizing different swimming modes. These species have comparable average lamellae (but reversed quantities across body regions) and precaudal vertebral counts, further supporting that the regional distribution of lamellae, centra morphometrics, and corpora calcera geometry are integral to differentiating vertebral column mechanics across thunniform and carangiform swimmers.

Within the geometric morphometric PCA, sand tiger data aligned with positie PC1 values; Carchariidae centra had expanded ventral intermediale and compressed dorsal intermediale sectors (Figure 9). All but two centra aligned with negative PC2 space, correlating with longer centra and more concave cones. The sand tiger and porbeagle overlapped in the morphospace, mirroring our mineral data PCA, and may characterize the mineral architecture and centra shape of shorter vertebral columns. An interesting finding is the disparity between the sand tiger and common thresher shark despite their shared carangiform swimming mode and heterocercal tail shape. While the common thresher shark tail is extremely elongated, the heterocercal shape is more comparable to the sand tiger than the lamnid's lunate tail. A recent study found that the shape of the caudal fin and the underlying musculoskeletal morphology (centra, musculature, and arches combined) are strongly correlated in 2D geometric morphometrics (Song & Lindgren, 2026). While we did not examine centra from within the caudal fin, it is plausible that the centrum morphology would correlate with tail size and shape. In the context of the main body, our findings suggest centra mineralization is independent of caudal fin shape and correlates more closely to variables impacting vertebral column mechanics.

Between the sand tiger specimens, the larger shark had centra with more lamellae and fewer nodes than the smaller specimen (Figure S5). We found an increase in lamellae quantity with fork length in the lamnids, and a similar trend for Carchariidae suggests that regardless of swimming style, centra will develop more lamellae to support the mechanics of swimming with a larger body. The decreasing nodes in relation to fork length in Carchariidae may reflect the spatial cue for mineralization—more lamellae within centra impose spatial constraints, limiting node quantity with increasing body size.

For family Carchariidae, the centrum (cylindrical) volume was not significant, but the mineral volume was significant in relation to fork length (Figure 10). Interestingly, the smaller sand tiger had larger mineralized volumes than the larger specimen, which was sourced from a managed care facility. While our observations may represent natural variation in centra morphology, this individual may also reflect mineralization influenced by environmental variables. In the context of Order Lamniformes, the mineral volumes for both sand tigers were within the range of volumes quantified for all sharks examined.

### 4.3 Common thresher shark (family Alopiidae)

Common thresher shark centra morphometrics followed a similar trend to the lamnid species; centra were small in the anterior region, increased in the middle body, and decreased in the posterior body region (Table S2, Figure 4). Standardized to fork length across species, the common thresher shark had the smallest anterior centra (width, height, and length) and the tallest and widest middle and posterior centra. The standardized centrum length in the middle and posterior regions was comparable in the common thresher shark to the lamnids, with posterior centra being shorter than middle body centra. The wide and tall centra have more surface area contact between adjacent intervertebral capsules, decreasing angular displacement between consecutive centra, and the cranio‐caudally compressed posterior centra limit the degree of flexion. Together, these factors create a stable vertebral column in the common thresher shark, and similar patterns in vertebral shape and flexibility of the column have been described for ichthyosaurs and cetaceans (Buchholtz, 2001; Long et al., 1997; Motani & McGowan, 1996).

In family Alopiidae, taller and wider centra have more lamellae but fewer nodes (Table S3, Figure S1). This finding may reflect mineralization patterns if lamellae are cued by spatial limitations—centra with more lamellae decrease the distance between neighboring lamellae and impose spatial limitations on node formation (Morse et al., 2022). However, we observed considerable intraspecific variation in node counts across common thresher shark specimens, and the thickness of lamellae would alter spatial boundaries, impacting this relationship. We did not measure lamellae thickness, but previous micro‐CT observations described highly variable lamellar thickness for the shortfin mako, white shark, and common thresher shark (Morse et al., 2022).

Knaub et al. (2024) quantified lamellae in common thresher sharks and defined anterior vertebrae as those with a neural arch and lateral processes; here, we defined anterior vertebrae as cranial to the insertion of the first dorsal fin (Knaub et al., 2024). The vertebrae quantified in Knaub et al., 2024, would align with the middle region in this data set and are defined as the vertebrae immediately below the first dorsal fin. With the addition of two adult common thresher shark specimens, we found that the number of lamellae across three body regions increased dramatically, in some cases doubling, from anterior to middle and posterior regions (Table S4, Figure 5e). The increased quantity of lamellae in common thresher sharks, particularly the highly elevated counts in middle and posterior regions, supports the functional dependence on the elongated caudal fin for both swimming and tail‐whipping, where the body, and likely the vertebral column, is subjected to extreme axial bending (Aalbers et al., 2010; Compagno, 1984, 1998; Hanan et al., 1993; Oliver et al., 2013; Smith et al., 2008).

The common thresher shark had the highest node average of all species, with a considerable range of node counts in the middle and posterior regions, while quantity was more consistent in anterior centra (Figure 5e). Knaub et al. (2024) examined juvenile and adult common thresher sharks and reported greater node counts in middle vertebrae than posterior vertebrae and in adults compared to juveniles (Knaub et al., 2024). Here, we found no node count difference in middle and posterior vertebrae in only adult specimens. Considering the results of the prior study and data from adult sharks presented here that include a body region anterior to the first dorsal fin, we have more evidence that nodes provide structural support. The nodes observed in common thresher shark centra likely provide stability to the numerous lamellae and distribute strain across the centrum during the high amplitude bending of tail‐whipping. We observed the greatest variation in counts in the middle and posterior body regions, suggesting nodes may vary intraspecifically. Thresher sharks have been documented using both overhead and sideways whipping behaviors, and individual common thresher sharks may develop more nodes with an increased reliance on a preferred type of tail‐whipping (Aalbers et al., 2010; Compagno, 1984, 1998; Hanan et al., 1993; Oliver et al., 2013; Smith et al., 2008).

Despite the high counts of lamellae and nodes in common thresher shark centra, our results suggest there is no relationship between the quantities of these structures (Table 4, Figure 6). Interestingly, a distinct cluster within the regression aligns with lower node counts that the other common thresher shark centra. These data are from the smallest common thresher shark in our study and may indicate a relationship between growth and node development. Alternatively, the cluster could be further evidence for the intraspecific variation in structures within family Alopiidae.

Like the lamellae and node regression, there appears to be no relationship between lamellae count and the proportion of mineral for the common thresher shark (Table 4, Figure S2). From the spread of data within the regression, centra seem to naturally divide by body regions, further demonstrating the increase in lamellae along the vertebral column. The comparable mineral proportion across body regions in common thresher shark centra may indicate that structures accommodate for the difference in overall volume across centrum sizes. For example, the posterior centra are smaller than those from the middle body, but make up the difference with a greater quantity of lamellae. Additionally, shark centra are stiffer when compressed at faster strain rates (Ingle et al., 2018; Porter et al., 2007). The vertebral column of thresher sharks undergoes rapid bending during tail‐whipping events (Oliver et al., 2013), and the speed of loading would stiffen centra and ultimately the axial body, without the need for additional mineral.

The common thresher shark had the largest average double cone angles and smallest average intermedialia angles, resulting in cranio‐caudally compressed centra with a shallow cone surface (Figure 7e). Likely, the consecutive shortened centra and shallow cones flatten the intervertebral capsules, limiting posterior deflection and reducing excessive flexion. The double cone and intermedialia angles are the most extreme in posterior centra, further flattening the capsule near the caudal fin. This would be advantageous for tail‐whipping behaviors, allowing for more controlled axial movement and fast, efficient strikes.

Common thresher shark centra had significantly smaller arches in the middle and posterior body, which allows for larger lateral intermedialia sectors (Figure S3e). While we did not directly quantify lamellae and nodes by individual sectors, we assume larger sectors would have a greater quantity of structures, dedicating a greater portion of each centrum to stress distribution in various planes. While it is assumed that common thresher sharks experience stress in the dorsoventral plane from overhead tail‐whips, they have been observed to perform sideways slaps that may load vertebral centra in various directions (Aalbers et al., 2010; Oliver et al., 2013). Additionally, prior examination of shark tail morphology described noticeably different ceratotrichia in the common thresher shark compared to other species (Crofts et al., 2019). The common thresher shark ceratotrichia are found in three compartments; the first runs along the long axis over the neural spines, the second overlaps the height of neural spines, and the third lies deep to the muscle along vertebral centra (Crofts et al., 2019). The first compartment is hypothesized to bear the tensile loads of tail‐whipping and may alter the morphology of the neural arch insertion in this species (Crofts et al., 2019). The position and orientation of the third compartment of ceratotrichia may be structurally related to the enlarged lateral intermedialia sectors in the posterior vertebrae.

Within the mineral PCA morphospace, middle and posterior common thresher shark centra were found exclusively on the negative side of the PC1 axis but were spread across the entirety of the PC2 axis (Figure 8). The numerous lamellae and centrum morphometrics of middle and posterior centra are most likely an adaptation for the tail‐whipping behavior of family Alopiidae considering these regions undergo immense strain and tension during extreme axial bending. The anterior centra were distinct from the other body regions and aligned with posterior centra from other species. Across all lamniformes, the body region ellipses increased in size from anterior to posterior regions and overlapped considerably. The increasing degree of variation (based on ellipse size) in centra morphology along the vertebral column may indicate a highly adaptive posterior region in lamniform sharks to support species‐specific swimming needs.

Family Alopiidae formed a distinct cluster in the geometric morphometric PCA and aligned with positive PC2 values due to the cranio‐caudally compressed centra with shallow cone surfaces (Figure 9). Common thresher sharks have a high precaudal vertebral count (average across specimens of 111), and the high number of short centra results in numerous yet short intervertebral capsules (Springer & Garrick, 1964). The short capsules would reduce flexion between adjacent centra but allow for more bending points and increase maximum curvature of the vertebral column (Brainerd & Patek, 1998). The position of common thresher shark centra along the PC1 axis is associated with the expansion of the dorsal intermediale and reduction in the ventral intermediale sector. This trend was also seen in family Lamnidae, but it is more extreme in family Alopiidae. During overhead tail‐whipping, the dorsal intermediale would experience loading conditions specific to dorsoventral bending, unlike the lateral loading during swimming. During the strike phase, the axial body is extended dorsally to move the caudal fin overhead (Oliver et al., 2013), and common thresher shark centra may redistribute mineral to the dorsal sector to withstand the mechanics of overhead tail‐whips. Additionally, within a single common thresher shark, the posterior centra may have a larger dorsal sector than other body regions to provide additional support to their distinct layered organization of ceratotrichia within the caudal fin (Crofts et al., 2019).

Family Alopiidae showed significant correlations between fork length and structure quantity (Figure S5). The negative relationship with lamellae count was unexpected, considering the other two families had positive correlations. Common thresher shark centra had comparable mineral proportion across centra regardless of lamellae quantity, and therefore increasing the number of lamellae with body size may provide little to no benefit to centra. Rather, centra may have a greater reliance on stress distribution to support tail‐whipping behaviors and develop more nodes with increasing body size rather than lamellae. This idea is further supported by our significant and positive correlation between fork length and node count. Within the radial mineral arrangement of shark centra, consistent lamellae counts and more nodes would create more “spokes” or mineral offshoots. Additional mineralized spokes within centra may have a similar function and behavior to spokes of elasmobranch tesserae which reinforce regions of collision between adjacent tesserae (Jayasankar et al., 2020; Seidel et al., 2016, 2019, 2020). If true, increasing node quantity in the common thresher shark would be integral to withstand loading regimes that would increase with fork length; adult thresher sharks need to bend a larger axial body and a proportionally larger caudal fin during tail‐whipping behaviors (Knaub et al., 2024).

Our regression of mineral volume and fork length for family Alopiidae suggests centra are proportionally smaller in larger sharks (Figure 10). We found that common thresher sharks do not deposit additional lamellae with increasing body size; rather, they bifurcate to add nodes. The additional nodes likely contribute very little to the overall mineral volume. Additionally, our regression does not account for the number of centra within the vertebral column. Common thresher sharks have the highest precaudal vertebral counts of all lamniform sharks examined; however, the centra are extremely short. This trade‐off may have influenced our regression trend: centra are proportionally smaller compared to shark body size, but within the entire shark, mineral is being deposited over a greater number of vertebrae along the column.

### 4.4 Basking shark (family Cetorhinidae)

Basking shark vertebrae are difficult to obtain due to the limited sampling opportunities from infrequent strandings. Additionally, the size of the vertebrae presents a long‐term storage challenge; our basking shark samples were three to five times larger than other vertebral samples (Table S2). Our sampling for this species was limited to one shark and we did not have vertebrae representing the posterior body region from our specimen.

Centra were larger in the anterior region compared to the middle region. In the mid‐body, width and length decreased while height was consistent with anterior centra (Table S2). Despite centrum size difference across species, the generalized Procrustes analysis of the landmark data removes the influence of size. Basking shark centra occupied the opposite end of the morphospace from the common thresher shark, correlating with elongated vertebral centra with a highly concave cone, a smaller dorsal intermediale sector, and a wider ventral intermediale sector (Figure 9). Considering regional trends observed in the other lamniformes, we would expect basking shark posterior centra to have proportionally larger lateral intermedialia sectors and occupy negative PC values. Cross‐sectional photos of basking shark vertebrae from the literature agree with our predictions and the morphology described here (Natanson et al., 2008; Parker & Stott, 1965).

Basking sharks had the largest intermedialia angles (66.6° average) and smallest double cone angles (105.8° average) of all species. These angles create an extreme concavity of the cone surface, resulting in a deep intervertebral joint. This may compensate for the fewer joints along the column, considering precaudal vertebral counts for basking sharks are lower than all other lamniformes examined here, ranging between 50 and 55 (Natanson et al., 2008; Springer & Garrick, 1964). Despite large intervertebral capsules, we expect that fewer precaudal vertebrae and elongated centra would create a less flexible column based off trends observed in bony fishes (Brainerd & Patek, 1998). This morphology may create a rigid vertebral column and be optimal for swimming performance in basking sharks, considering they experience large wave drag forces while filter‐feeding just below the surface (Compagno, 1984; Hertel, 1966).

Our findings corroborate previous work describing basking shark vertebral morphology that differs from other lamniform sharks, especially internal architecture (Hasse, 1879; Natanson et al., 2008; Ridewood, 1921). We were unable to include basking shark centra in our mineral data analysis due to the lack of well‐defined lamellae and nodes (Figure 1). Basking shark centra do exhibit peripheral radial lamellae, however, even in adult specimens, the structures are poorly calcified (Kyle, 1927; Owen, 1866; Parker & Stott, 1965; Sadowsky, 1973). Basking sharks are one of few planktivorous shark species, and the relationship between diet, environmental conditions, and vertebral structure formation (e.g., lamellae) is not fully understood (Campana et al., 2002; Kalish & Johnston, 2001; Kerr et al., 2006; MacNeil et al., 2005; Smith et al., 2013, 2016). Our specimen (728.3 cm FL) was considered an adult but lacked sufficient calcification to distinguish mineralized structures for quantification. However, like other lamniformes, the basking shark is known to exhibit breaching behaviors (jumping almost entirely or completely above the surface of the water) (Klimley et al., 2024). Breaching requires animals to reach high swimming speeds and generate high power output, and basking sharks can exhibit the same velocity as white sharks during breaching events (Johnston et al., 2018). It is possible that the presence of radial lamellae (although poorly calcified) in combination with the cone morphology of basking shark vertebrae contributes to the immense power generation for breaching, however, further examination would be necessary.

## 5 CONCLUSIONS

In comparison to bone and other cartilages (hyaline and tessellated), the 3D morphological variation of areolar mineralization is poorly studied (Dean et al., 2015; Seidel et al., 2020). The data presented here provide a robust foundation for exploring skeletal adaptations in sharks, specifically the functional morphology of mineralized, cartilaginous vertebrae across natural phylogenetic variation. Our findings highlight that lamniform shark vertebral morphology is governed by several factors, including centrum size, mineral amount, and structure organization. Regional variation along the column appears to adapt in support of increased flexibility or stiffness, depending on swimming strategy. The arrangement and quantity of mineral structures (lamellae and nodes) vary among lamniform shark families and may serve different yet complementary functions contributing to centra mechanics. A broader trade‐off between the shape of the mineralized corpora calcera, vertebral number, and intervertebral joint morphology—though not directly examined in our study—is likely a crucial factor in altering whole vertebral column mechanics, and further study of joint morphology is warranted. Additionally, scaling relationships between mineral and shark length suggest that shark centra may adapt internal architecture at larger body sizes rather than contributing to overall vertebral size. While this work was limited to sharks of the order Lamniformes, comparative studies across shark orders (e.g., Carcharhiniformes) could help contextualize lamniform adaptations. Additionally, we suggest future work to incorporate finite‐element analysis to examine the reported mechanical properties of shark vertebrae in the context of their morphology described in this study. Overall, our findings contribute to the understanding of vertebrate skeletal tissues and their structural diversity.

## AUTHOR CONTRIBUTIONS

**Jamie L. Knaub:** Concept and design, acquisition of data, data analysis and interpretation, drafting of manuscript, critical revision of manuscript, and approval of article. **Madisan Biordi:** Concept and design, acquisition of data, data analysis and interpretation, and approval of article. **Emma Pawlik:** Concept and design, acquisition of data, data analysis and interpretation, and approval of article. **Michelle Passerotti:** Resources, critical revision of manuscript, and approval of article. **Lisa J. Natanson:** Resources, critical revision of manuscript, and approval of article. **Tricia Meredith:** Resources, critical revision of manuscript, and approval of article. **Marianne Porter:** Concept and design, data analysis and interpretation, resources, editing of early paper drafts, critical revision of manuscript, and approval of article.

## Supporting information


**Table S1.** Shark specimen source information. A source method is listed for each specimen and was categorized as one of the following: stranding, managed care, sport fishing (includes tournaments and recreational fishing), or commercial longline. A link to each specimen's homepage on MorphoSource (MS) is included.
**Table S2:** Average centrum morphometrics by species. Centrum width, height, and length (in millimeters) are presented as an average of all sharks of the respective species (adult sharks only) and standardized to fork length (in italics below).
**Table S3:** Mineral structures (lamellae and node counts) and standardized centrum morphometrics log‐transformed linear regression model results.
**Table S4:** Mineral data averages by species across body regions (including standard deviations). An average (mean of all body regions) is provided for each mineral variable by species in bolded italics below.
**Table S5:** Mineral data PCA proportion of variance and loadings. Centrum morphometrics (width, length, and height) were standardized to individual fork length.
**Table S6:** Mineral structures (lamellae and node counts) and fork length log‐transformed linear regression model results.
**Figure S1:** Log‐transformed linear regressions for mineral structures and standardized centrum morphometrics. Regression models for lamellae were significant for all morphometrics: (a) width (*F*_1,135_ = 18.050; *p* < 0.001), (c) height (*F*_1,135_ = 26.580; *p* < 0.001), and (e) length (*F*_1,135_ = 20.880; *p* < 0.001). Wider (*y* = 0.844*x* + 4.691; *R* ^2^ = 0.111) and taller (*y* = 1.035*x* + 5.038; *R* ^*2*^ = 0.158) centra are predicted to have more lamellae. Longer centra (*y* = −0.746*x* + 1.148; *R* ^2^ = 0.128) are predicted to have fewer lamellae. Regressions for node count were not significant for (b) width, (d) height, and (f) length. Point shape depicts body region and color represents species. Relationships for all sharks are shown as a black line and family‐specific relationships are depicted by color regression lines (Alopiidae in orange, Carchariidae in pink, and Lamnidae in teal). The shaded gray ribbon for each line represents the 95% confidence interval.
**Figure S2:** Log‐log regression of lamellae count and proportion of mineral. The relationship for all species (black line) was significant (*F*_1,135_ = 9.486; *p* = 0.003). Centra with more lamellae are predicted to have more mineral (*y* = 0.135*x* – 1.54; *R* ^*2*^ = 0.059). Point shape depicts body region and color represents species. Family‐specific relationships are depicted by color regression lines (Alopiidae in orange, Carchariidae in pink, and Lamnidae in teal). The shaded gray ribbon for each line represents the 95% confidence interval.
**Figure S3:** Dorsal and ventral arch angles across body regions for 5 lamniform species (a–c: lamnids, d, e: non‐lamnids). Violin colors increase in saturation across body regions for each species. Violin width depicts distribution of points, and a boxplot within each violin indicates quartile ranges. The median and mean are shown as a black horizontal line and red point, respectively. Tukey post hoc results are shown above each violin and indicate significant differences. N values represent the number of individual sharks examined for each species. The number of vertebrae examined for each region can be found in Table 1.
**Figure S4:** Landmark variance plots for the 19 specimens (53 centra) used in the geometric morphometric analysis. 3D renderings of centra (top) depict landmark placement for the anterior cone face (warmer colors), posterior cone face (cooler colors) and the cone apex (green). Individual lamellae and nodes were unable to be landmarked due to their analogous position across centra. The landmarks selected for the level of the cone apex were designed to capture shape change of the intermedialia via four sectors (outlined in green). Individual landmarks are displayed in a morphospace below to visualize the spread of points and overall variation. (a) External side view of landmark placement. (b) Top‐down view with anterior cone removed to visualize internal landmark placement and variation. See footnote in Table 1 for details on specific vertebrae used in the geometric morphometric analysis.
**Figure S5:** Log‐transformed linear regressions for fork length and (a) lamellae count, and (b) node count. Regression models for all species (black line) were significant for (a) lamellae (*F*_1,135_ = 16.010; *p* < 0.001), but not for (b) nodes. Longer sharks are predicted to have less lamellae (*y* = −1.874*x* + 7.572; *R* ^*2*^ = 0.099). Point shape depicts body region and color represents species. Family‐specific relationships are depicted by color regression lines (Alopiidae in orange, Carchariidae in pink, and Lamnidae in teal). The shaded gray ribbon for each line represents the 95% confidence interval.

## Data Availability

Reconstructed micro‐CT scans of vertebrae and associated metadata are available from MorphoSource (https://doi.org/10.17602/M2/L812881). The data that support the findings of this study are available from the corresponding author upon reasonable request.
