# Supplementary material for: Skeletons of swiftly swimming sharks: Three‐dimensional analysis of lamniform vertebral morphology and mineral architecture
Source: J Anat. 2026 Jul 14:10.1111/joa.70209. Online ahead of print. doi: 10.1111/joa.70209 (PMC13398450; doi:10.1111/joa.70209)
Supplement: Supplementary file 1 — Table S1. Shark specimen source information. A source method is listed for each specimen and was categorized as one of the following: stranding, managed care, sport fishing (includes tournaments and recreational fishing), or commercial longline. A link to each specimen's homepage on MorphoSource (MS) is included. Table S2: Average centrum morphometrics by species. Centrum width, height, and length (in millimeters) are presented as an average of all sharks of the respective species (adult sharks only) and standardized to fork length (in italics below). Table S3: Mineral structures (lamellae and node counts) and standardized centrum morphometrics log‐transformed linear regression model results. Table S4: Mineral data averages by species across body regions (including standard deviations). An average (mean of all body regions) is provided for each mineral variable by species in bolded italics below. Table S5: Mineral data PCA proportion of variance and loadings. Centrum morphometrics (width, length, and height) were standardized to individual fork length. Table S6: Mineral structures (lamellae and node counts) and fork length log‐transformed linear regression model results. Figure S1: Log‐transformed linear regressions for mineral structures and standardized centrum morphometrics. Regression models for lamellae were significant for all morphometrics: (a) width (F 1,135 = 18.050; p < 0.001), (c) height (F 1,135 = 26.580; p < 0.001), and (e) length (F 1,135 = 20.880; p < 0.001). Wider (y = 0.844x + 4.691; R 2 = 0.111) and taller (y = 1.035x + 5.038; R 2 = 0.158) centra are predicted to have more lamellae. Longer centra (y = −0.746x + 1.148; R 2 = 0.128) are predicted to have fewer lamellae. Regressions for node count were not significant for (b) width, (d) height, and (f) length. Point shape depicts body region and color represents species. Relationships for all sharks are shown as a black line and family‐specific relationships are depicted by color regression lines (Alo [file JOA-9999-0-s001.docx]

**Supplementary Table 1:** Shark specimen source information. A source method is listed for each specimen and was categorized as one of the following: stranding, managed care, sport fishing (includes tournaments and recreational fishing), or commercial longline. A link to each specimen’s homepage on MorphoSource (MS) is included.

| Species | Fork length (FL, cm) | Sex | Method | Location | Year | MS Link |  |
| --- | --- | --- | --- | --- | --- | --- | --- |
| Basking shark (*C. maximus*) | | 728.3 | F | Stranding | Rhode Island | 2013 | [BA18](https://www.morphosource.org/concern/biological_specimens/000765446?locale=en) |
| Sand tiger | 190.5† | M | Managed care | California | 2024 | [SndTg48318](https://www.morphosource.org/concern/biological_specimens/000765665?locale=en) |  |
| *(C. taurus)* | 221.0 | F | Stranding | Rhode Island | 2024 | [SndTg221](https://www.morphosource.org/concern/biological_specimens/000765776?locale=en) |  |
| White shark | 322.6 | M | Stranding | Massachusetts | 2016 | [W153](https://www.morphosource.org/concern/biological_specimens/000765592?locale=en) |  |
| *(C. carcharias)* | 331.0 | M | Stranding | Massachusetts | 2015 | [W150](https://www.morphosource.org/concern/biological_specimens/000765579?locale=en) |  |
|  | 377.0 | M | Stranding | Massachusetts | 2012 | [W145](https://www.morphosource.org/concern/biological_specimens/000765426?locale=en) |  |
|  | 380.9 | M | Stranding | Massachusetts | 2015 | [W149](https://www.morphosource.org/concern/biological_specimens/000765484?locale=en) |  |
| Shortfin mako | 182.8 | M | Sport fishing | New York | 2011 | [M508](https://www.morphosource.org/concern/biological_specimens/000697466?locale=en) |  |
| *(I. oxyrinchus)* | 260.4 | M | Sport fishing | Rhode Island | 2005 | [M494](https://www.morphosource.org/concern/biological_specimens/000752621?locale=en) |  |
|  | 291.5 | F | Sport fishing | New York | 2016 | [M594](https://www.morphosource.org/concern/biological_specimens/000697562?locale=en) |  |
|  | 292.1 | F | Sport fishing | Florida | 2015 | [M527](https://www.morphosource.org/concern/biological_specimens/000697513?locale=en) |  |
|  | 294.6 | F | Sport fishing | Florida | 2015 | [M528](https://www.morphosource.org/concern/biological_specimens/000697534?locale=en) |  |
| Porbeagle  *(L. nasus)* | 224.4 | F | Sport fishing | Rhode Island | 2015 | [LN644](https://www.morphosource.org/concern/biological_specimens/000764558?locale=en) |  |
|  | 225.7 | F | Sport fishing | Rhode Island | 2014 | [LN639](https://www.morphosource.org/concern/biological_specimens/000764523?locale=en) |  |
|  | 234.0 | F | Sport fishing | Maine | 2011 | [LN636](https://www.morphosource.org/concern/biological_specimens/000752425?locale=en) |  |
|  | 241.0 | F | Sport fishing | Rhode Island | 2015 | [LN643](https://www.morphosource.org/concern/biological_specimens/000764545?locale=en) |  |
|  | 247.6 | F | Sport fishing | Maine | 2012 | [LN633](https://www.morphosource.org/concern/biological_specimens/000752443?locale=en) |  |
|  | 248.0 | F | Commercial longline | Grand Banks, N. Atlantic | 2016 | [LN650](https://www.morphosource.org/concern/biological_specimens/000764602?locale=en) |  |
| Common | 194.6 | M | Stranding | Massachusetts | 2022 | [CT194](https://www.morphosource.org/concern/biological_specimens/000766000?locale=en) |  |
| thresher shark | 208.0 | M | Sport fishing | New York | 2017 | [CTSIYC](https://www.morphosource.org/concern/biological_specimens/000582961?locale=en) |  |
| *(A. vulpinus)* | 209.9 | M | Sport fishing | Rhode Island | 2014 | [CT366](https://www.morphosource.org/concern/biological_specimens/000582838?locale=en) |  |
|  | 226.0 | M | Stranding | Massachusetts | 2024 | [CT406](https://www.morphosource.org/concern/biological_specimens/000765898?locale=en) |  |
|  | 229.0 | F | Sport fishing | Rhode Island | 2016 | [CT376](https://www.morphosource.org/concern/biological_specimens/000582910?locale=en) |  |
|  | 241.9 | F | Sport fishing | Maine | 2014 | [CT369](https://www.morphosource.org/concern/biological_specimens/000582800?locale=en) |  |

† estimated FL

**Supplementary Table 2:** Average centrum morphometrics by species. Centrum width, height, and length (in millimeters) are presented as an average of all sharks of the respective species (adult sharks only) and standardized to fork length (in italics below).

| **Species** | **Combined regions** | | | **Anterior** | | | **Middle** | | | **Posterior** | | |
| --- | --- | --- | --- | --- | --- | --- | --- | --- | --- | --- | --- | --- |
|  | Width | Height | Length | Width | Height | Length | Width | Height | Length | Width | Height | Length |
| Shortfin mako | 37.04  *0.14* | 36.59  *0.14* | 17.31  *0.07* | 37.90  *0.14* | 37.31  *0.14* | 17.78  *0.07* | 41.23  *0.16* | 40.79  *0.15* | 19.82  *0.08* | 31.98  *0.12* | 31.68  *0.12* | 14.32  *0.06* |
| Porbeagle | 37.91  *0.16* | 37.45  *0.16* | 18.41  *0.08* | 38.47  *0.16* | 38.74  *0.16* | 17.67  *0.07* | 41.09  *0.17* | 41.21  *0.17* | 20.59  *0.09* | 34.16  *0.14* | 32.41  *0.14* | 16.98  *0.07* |
| White shark | 51.92  *0.15* | 53.10  *0.15* | 23.67  *0.07* | 53.17  *0.15* | 54.82  *0.16* | 24.95  *0.07* | 61.08  *0.17* | 61.98  *0.18* | 27.67  *0.08* | 38.04  *0.11* | 38.99  *0.11* | 16.62  *0.05* |
| Sand tiger | 34.26  *0.15* | 33.61  *0.15* | 19.46  *0.09* | 37.10  *0.17* | 35.95  *0.16* | 20.92  *0.09* | 35.68  *0.16* | 35.44  *0.16* | 19.64  *0.09* | 30.02  *0.14* | 29.43  *0.13* | 17.81  *0.08* |
| Common thresher shark | 36.03  *0.17* | 36.39  *0.17* | 13.91  *0.07* | 27.84  *0.12* | 30.19  *0.13* | 12.41  *0.06* | 38.79  *0.19* | 38.01  *0.18* | 16.24  *0.08* | 35.26  *0.17* | 36.23  *0.17* | 12.11  *0.06* |
| Basking shark | 157.06  *0.22* | 146.14  *0.20* | 104.86  *0.14* | 164.09  *0.23* | 145.53  *0.20* | 113.36  *0.16* | 150.03  *0.21* | 146.75  *0.20* | 96.36  *0.13* | NA  *NA* | NA  *NA* | NA  *NA* |

**Supplementary Table 3:** Mineral structures (lamellae and node counts) and standardized centrum morphometrics log-transformed linear regression model results.

| **Lamellae vs** | **Centrum width** | | | | **Centrum height** | | | **Centrum length** | | |
| --- | --- | --- | --- | --- | --- | --- | --- | --- | --- | --- |
| Intercept | | 4.691 | | | 5.038 | | | 1.148 | | |
| Slope | | 0.844 | | | 1.035 | | | -0.746 | | |
| All sharks | | *df* | *F* | *p* | *df* | *F* | *p* | *df* | *F* | *p* |
|  | | 1,135 | 18.050 | <.001 | 1,135 | 26.580 | <.001 | 1,135 | 20.880 | <.001 |
| *R^2^* | | 0.111 | | | 0.158 | | | 0.128 | | |
| By family | | *df* | *F* | *p* | *df* | *F* | *p* | *df* | *F* | *p* |
| Lamnidae | | 47 | 3.591 | 0.064 | 47 | 3.217 | 0.079 | 47 | 3.714 | 0.060 |
| *R^2^* | |  | 0.051 |  |  | 0.044 |  |  | 0.054 |  |
| Carchariidae | | 16 | 0.113 | 0.741 | 16 | 0.009 | 0.924 | 16 | 0.997 | 0.333 |
| *R^2^* | |  | -0.055 |  |  | -0.062 |  | -0.0001 | | |
| Alopiidae | | 68 | 19.000 | <.001 | 68 | 32.160 | <.001 | 68 | 0.131 | 0.719 |
| *R^2^* | |  | 0.207 |  |  | 0.311 |  |  | -0.013 |  |
|  | |  |  |  |  |  |  |  |  |  |
| **Nodes vs** | | **Centrum width** | | | **Centrum height** | | | **Centrum length** | | |
| Intercept | | 1.594 | | | 1.765 | | | 1.059 | | |
| Slope | | 0.108 | | | 0.201 | | | -0.126 | | |
| All sharks | | *df* | *F* | *p* | *df* | *F* | *p* | *df* | *F* | *p* |
|  | | 1,135 | 0.739 | 0.392 | 1,135 | 2.426 | 0.122 | 1,135 | 1.487 | 0.225 |
| *R^2^* | | -0.002 | | | 0.010 | | | 0.004 | | |
| By family | | *df* | *F* | *p* | *df* | *F* | *p* | *df* | *F* | *p* |
| Lamnidae | | 47 | 0.070 | 0.792 | 47 | 0.298 | 0.588 | 47 | 0.0004 | 0.984 |
| *R^2^* | |  | -0.020 |  |  | -0.015 |  |  | -0.021 |  |
| Carchariidae | | 16 | 0.053 | 0.820 | 16 | 0.002 | 0.962 | 16 | 0.005 | 0.945 |
| *R^2^* | |  | -0.059 |  |  | -0.062 |  |  | -0.062 |  |
| Alopiidae | | 68 | 13.800 | 0.0004 | 68 | 14.530 | <.001 | 68 | 4.183 | 0.045 |
| *R^2^* | |  | 0.157 |  |  | 0.164 |  |  | 0.044 |  |

**Supplementary Table 4:** Mineral data averages by species across body regions (including standard deviations). An average (mean of all body regions) is provided for each mineral variable by species in bolded italics below.

| **Variable** | **Shortfin mako** | | | **Porbeagle** | | | **White shark** | | | **Sand tiger** | | | **Common thresher shark** | | |
| --- | --- | --- | --- | --- | --- | --- | --- | --- | --- | --- | --- | --- | --- | --- | --- |
|  | Ant | Mid | Post | Ant | Mid | Post | Ant | Mid | Post | Ant | Mid | Post | Ant | Mid | Post |
| Lamellae | 17.13 ± 2.24 | 20.53 ± 2.36 | 20.27 ± 2.45 | 12.96 ± 1.94 | 15.83 ± 1.91 | 17.71 ± 1.63 | 17.75 ± 1.52 | 25.42 ± 3.51 | 28.34 ± 4.93 | 13.94 ± 1.83 | 13.22 ± 1.94 | 12.55 ± 0.62 | 19.47 ± 1.14 | 31.16 ± 3.24 | 36.75 ± 3.69 |
|  | **19.31 ± 2.35** | | | ***15.50* ± 1.83** | | | ***23.84* ± 3.32** | | | ***13.24* ± 1.46** | | | ***29.13* ± 2.69** | | |
| Nodes | 25.13 ± 7.12 | 23.53 ± 9.46 | 19.87 ± 6.89 | 10.29 ± 2.39 | 15.25 ± 6.74 | 10.33 ± 3.11 | 21.00 ± 3.35 | 21.25 ± 3.56 | 11.00 ± 1.73 | 30.78 ± 3.54 | 34.11 ± 2.91 | 35.50 ± 3.03 | 33.14 ± 2.55 | 38.38 ± 20.02 | 33.97 ± 8.85 |
|  | ***22.85* ± 7.82** | | | ***11.96* ± 4.08** | | | ***17.75* ± 2.88** | | | ***33.46* ± 3.16** | | | ***35.16* ± 10.47** | | |
| Double cone angle (°) | 132.58 ± 2.67 | 131.36 ± 3.65 | 133.38 ± 2.47 | 132.76 ± 5.89 | 126.22 ± 5.26 | 124.87 ± 2.14 | 133.95 ± 2.66 | 131.61 ± 3.30 | 135.61 ± 1.76 | 118.27 ± 3.55 | 119.39 ± 6.42 | 113.47 ± 1.90 | 132.65 ± 1.20 | 135.81 ± 3.91 | 141.24 ± 4.47 |
|  | ***132.44* ± 2.93** | | | ***127.95* ± 4.43** | | | ***133.72* ± 2.57** | | | ***117.04* ± 3.95** | | | ***136.57* ± 3.19** | | |
| Intermedialia angle (°) | 41.13 ± 2.55 | 43.54 ± 2.71 | 39.96 ± 3.16 | 41.76 ± 6.18 | 50.52 ± 4.72 | 50.37 ± 3.22 | 40.14 ± 2.80 | 41.73 ± 1.11 | 36.79 ± 2.93 | 49.74 ± 2.78 | 51.36 ± 7.32 | 56.34 ± 3.01 | 36.71 ± 0.96 | 36.56 ± 2.08 | 27.35 ± 2.27 |
|  | ***41.55* ± 2.81** | | | ***47.55* ± 4.71** | | | ***39.55* ± 2.28** | | | ***52.48* ± 4.37** | | | ***33.54* ± 1.77** | | |
| Dorsal arch angle (°) | 21.99 ± 3.51 | 18.54 ± 3.12 | 16.74 ± 2.57 | 29.24 ± 3.56 | 25.81 ± 1.24 | 24.11 ± 4.73 | 24.92 ± 1.77 | 22.87 ± 1.61 | 24.15 ± 5.88 | 26.32 ± 0.83 | 29.12 ± 2.30 | 29.00 ± 2.31 | 30.37 ± 2.06 | 19.34 ± 1.80 | 18.02 ± 2.09 |
|  | ***19.09* ± 3.07** | | | ***26.39* ± 3.18** | | | ***23.98* ± 3.09** | | | ***28.15* ± 1.81** | | | ***22.58* ± 1.98** | | |
| Ventral arch angle (°) | 20.23 ± 5.00 | 16.39 ± 1.90 | 15.81 ± 2.75 | 31.41 ± 2.70 | 28.18 ± 3.87 | 26.24 ± 3.08 | 23.61 ± 3.13 | 22.52 ± 1.88 | 23.20 ± 3.59 | 27.99 ± 1.37 | 30.09 ± 1.47 | 32.56 ± 0.70 | 34.61 ± 1.05 | 21.52 ± 1.57 | 23.28 ± 1.62 |
|  | ***17.48* ± 3.22** | | | ***28.61* ± 3.22** | | | ***23.11* ± 2.87** | | | ***30.21* ± 1.18** | | | ***26.47* ± 1.41** | | |

**Supplementary Table 5:** Mineral data PCA proportion of variance and loadings. Centrum morphometrics (width, length, and height) were standardized to individual fork length.

| **Variance** | **PC1** | **PC2** | **PC3** | **PC4** | **PC5** |
| --- | --- | --- | --- | --- | --- |
| Proportion of variance | 0.4727 | 0.2427 | 0.1166 | 0.1011 | 0.0292 |
| Standard deviation | 2.0626 | 1.4780 | 1.0245 | 0.9539 | 0.5125 |
| **Loading** |  |  |  |  |  |
| Centrum width | -0.2707 | 0.5322 | -0.0283 | -0.1733 | 0.1010 |
| Centrum height | -0.2947 | 0.4972 | -0.0994 | -0.2408 | 0.0313 |
| Centrum length | 0.1523 | 0.6124 | -0.0388 | 0.1383 | -0.0485 |
| Lamellae count | -0.4238 | -0.0925 | -0.1982 | 0.0103 | -0.7606 |
| Node count | -0.1262 | -0.0689 | -0.8641 | 0.3512 | 0.3082 |
| Dorsal arch angle (°) | 0.4119 | -0.0119 | -0.1975 | -0.4000 | 0.2028 |
| Ventral arch angle (°) | 0.3220 | -0.0309 | -0.3661 | -0.6243 | -0.3125 |
| Intermedialia angle (°) | 0.4174 | 0.2330 | 0.0867 | 0.3057 | -0.1407 |
| Double cone angle (°) | -0.4165 | -0.1609 | 0.1464 | -0.3552 | 0.3930 |

**Supplementary Table 6:** Mineral structures (lamellae and node counts) and fork length log-transformed linear regression model results.

| **Fork length vs** | | **Lamellae** | | | **Nodes** | | |
| --- | --- | --- | --- | --- | --- | --- | --- |
| Intercept | | 7.572 | | |  | 2.433 |  |
| Slope | | -1.874 | | |  | -0.049 |  |
| All sharks | | *df* | *F* | *p* | *df* | *F* | *p* |
|  | | 1,135 | 16.010 | <.001 | 1,135 | 3.833 | 0.052 |
| *R^2^* | | 0.099 | | |  | 0.020 |  |
| By family | *df* | *F* | *p* | *df* | *F* | *p* |  |
| Lamnidae | | 47 | 8.559 | 0.005 | 47 | 9.969 | 0.003 |
| *R^2^* | | 0.136 | | |  | 0.157 |  |
| Carchariidae | | 16 | 15.670 | 0.001 | 16 | 8.000 | 0.012 |
| *R^2^* | | 0.463 | | |  | 0.292 |  |
| Alopiidae | | 68 | 10.330 | 0.002 | 68 | 97.41 | <.001 |
| *R^2^* | | 0.119 | | |  | 0.583 |  |


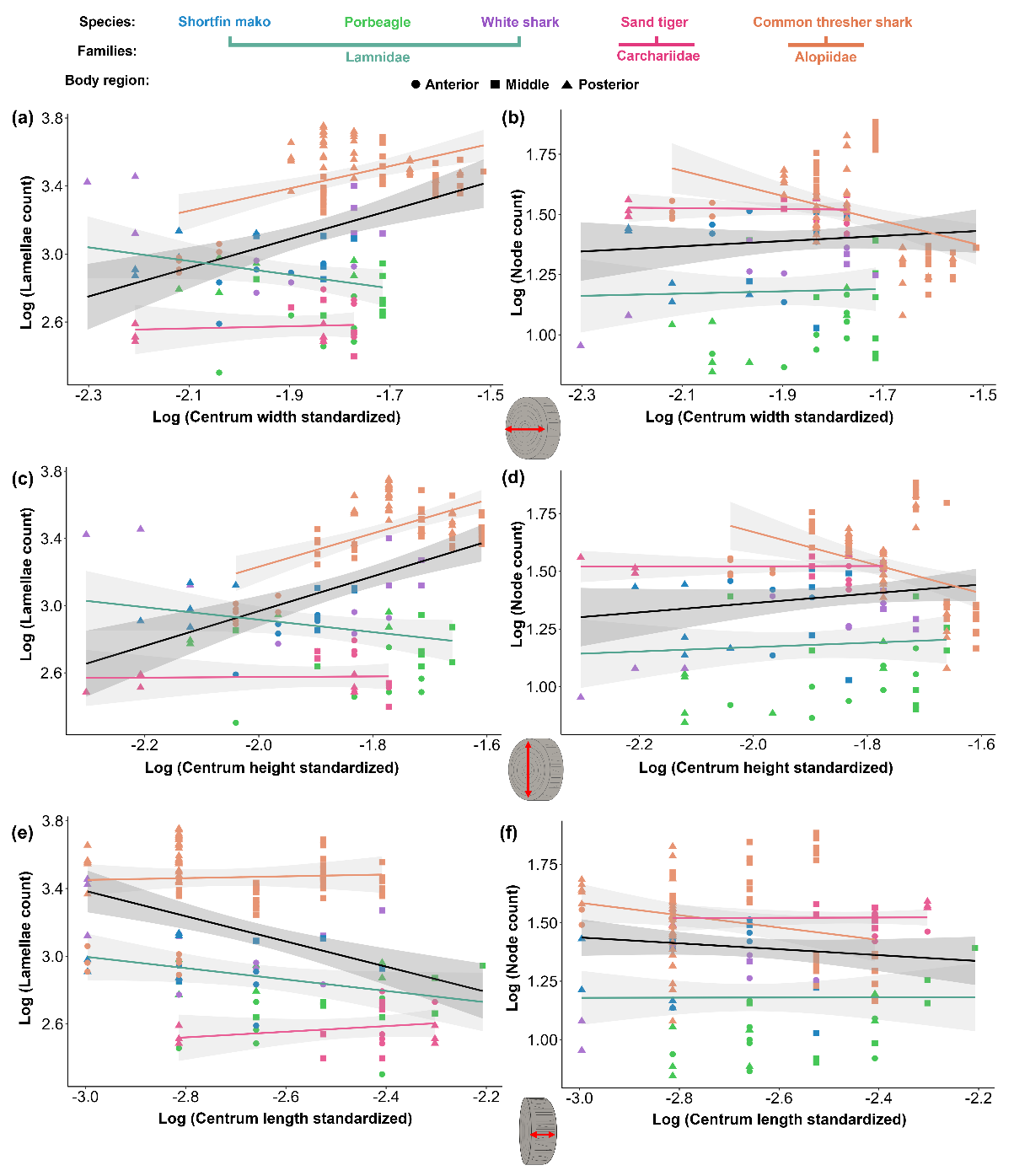


**Supplementary Figure 1:** Log-transformed linear regressions for mineral structures and standardized centrum morphometrics. Regression models for lamellae were significant for all morphometrics: (a) width (*F*_1,135_ = 18.050; *p*<0.001), (c) height (*F*_1,135_ = 26.580; *p*<0.001), and (e) length (*F*_1,135_ = 20.880; *p*<0.001). Wider (*y* = 0.844*x* + 4.691; *R^2^* = 0.111) and taller (*y* = 1.035*x* + 5.038; *R^2^* = 0.158) centra are predicted to have more lamellae. Longer centra (*y* = -0.746*x* + 1.148; *R^2^* = 0.128) are predicted to have fewer lamellae. Regressions for node count were not significant for (b) width, (d) height, and (f) length. Point shape depicts body region and color represents species. Relationships for all sharks are shown as a black line and family-specific relationships are depicted by color regression lines (Alopiidae in orange, Carchariidae in pink, and Lamnidae in teal). The shaded gray ribbon for each line represents the 95% confidence interval.


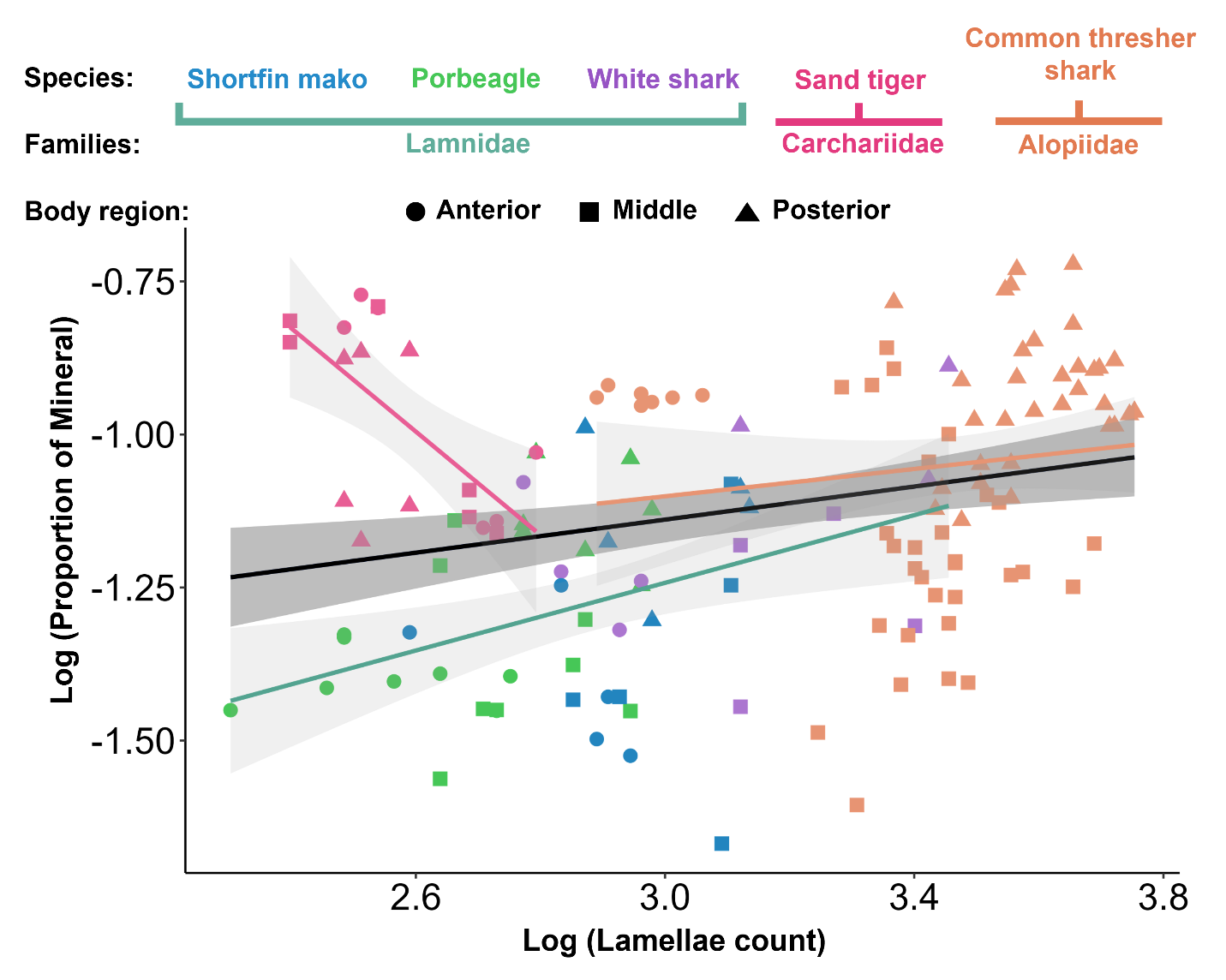


**Supplementary Figure 2:** Log-log regression of lamellae count and proportion of mineral. The relationship for all species (black line) was significant (*F*_1,135_ = 9.486; *p*=0.003). Centra with more lamellae are predicted to have more mineral (*y* = 0.135*x* – 1.54; *R^2^* = 0.059). Point shape depicts body region and color represents species. Family-specific relationships are depicted by color regression lines (Alopiidae in orange, Carchariidae in pink, and Lamnidae in teal). The shaded gray ribbon for each line represents the 95% confidence interval.


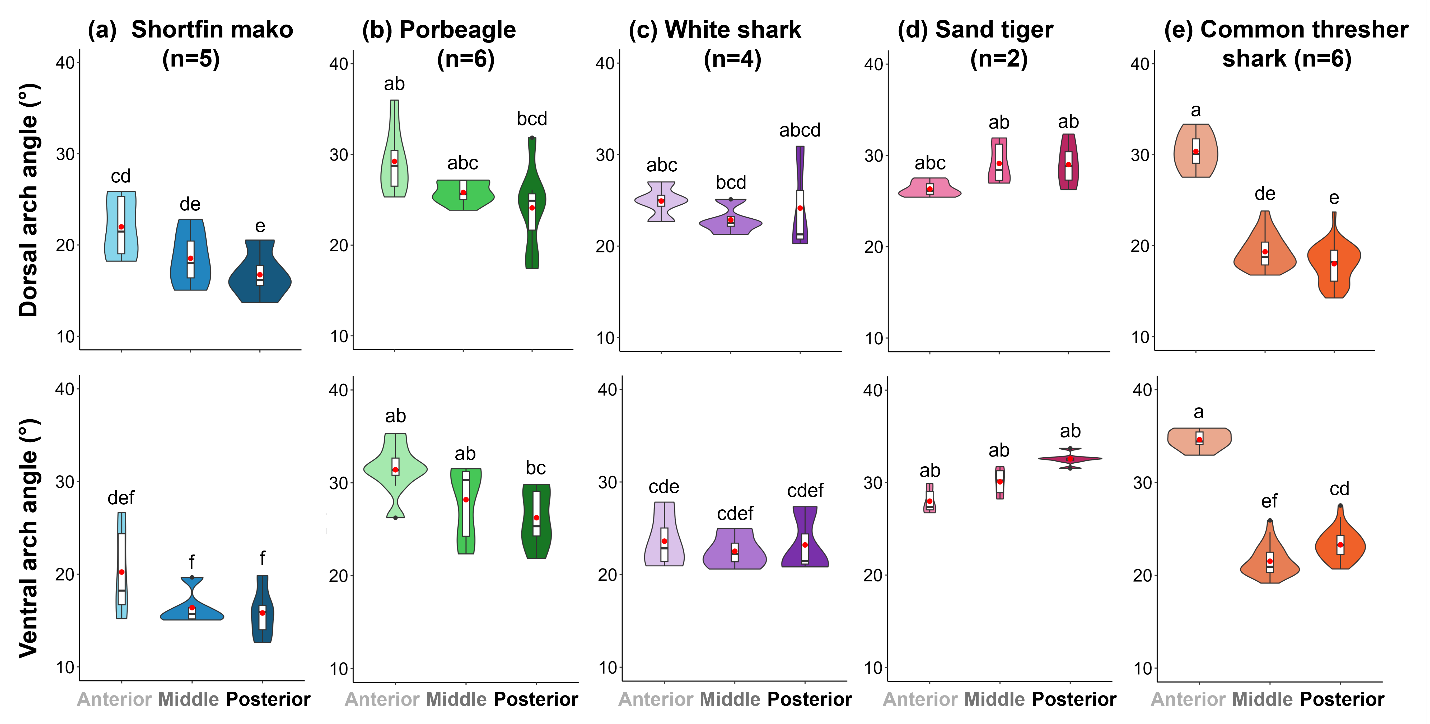


**Supplementary Figure 3:** Dorsal and ventral arch angles across body regions for 5 lamniform species (a-c: lamnids, d,e: non-lamnids). Violin colors increase in saturation across body regions for each species. Violin width depicts distribution of points, and a boxplot within each violin indicates quartile ranges. The median and mean are shown as a black horizontal line and red point, respectively. Tukey *post hoc* results are shown above each violin and indicate significant differences. N values represent the number of individual sharks examined for each species. The number of vertebrae examined for each region can be found in Table 1.


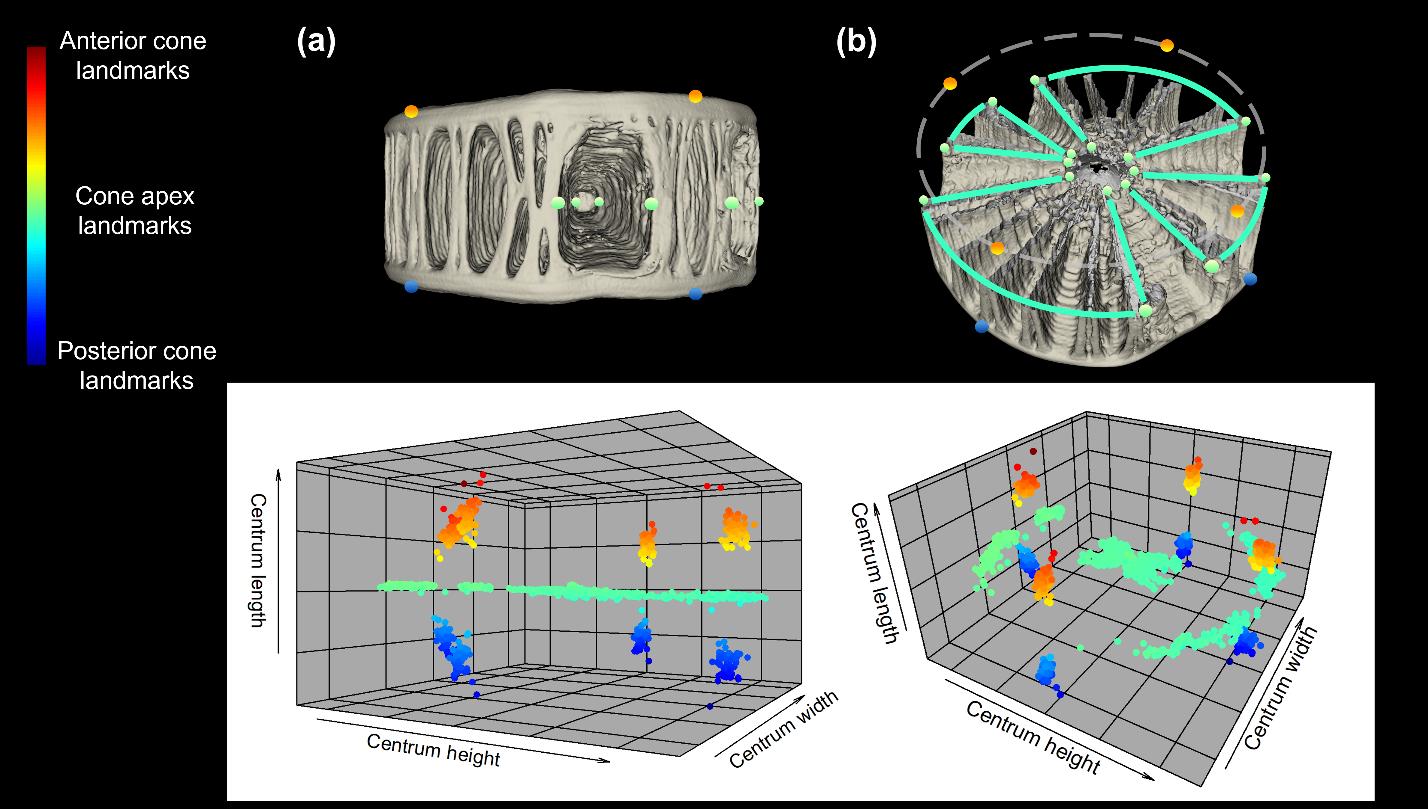


**Supplementary Figure 4:** Landmark variance plots for the 19 specimens (53 centra) used in the geometric morphometric analysis. 3D renderings of centra (top) depict landmark placement for the anterior cone face (warmer colors), posterior cone face (cooler colors) and the cone apex (green). Individual lamellae and nodes were unable to be landmarked due to their analogous position across centra. The landmarks selected for the level of the cone apex were designed to capture shape change of the intermedialia via four sectors (outlined in green). Individual landmarks are displayed in a morphospace below to visualize the spread of points and overall variation. (a) External side view of landmark placement. (b) Top-down view with anterior cone removed to visualize internal landmark placement and variation. See footnote in Table 1 for details on specific samples used in the geometric morphometric analysis.


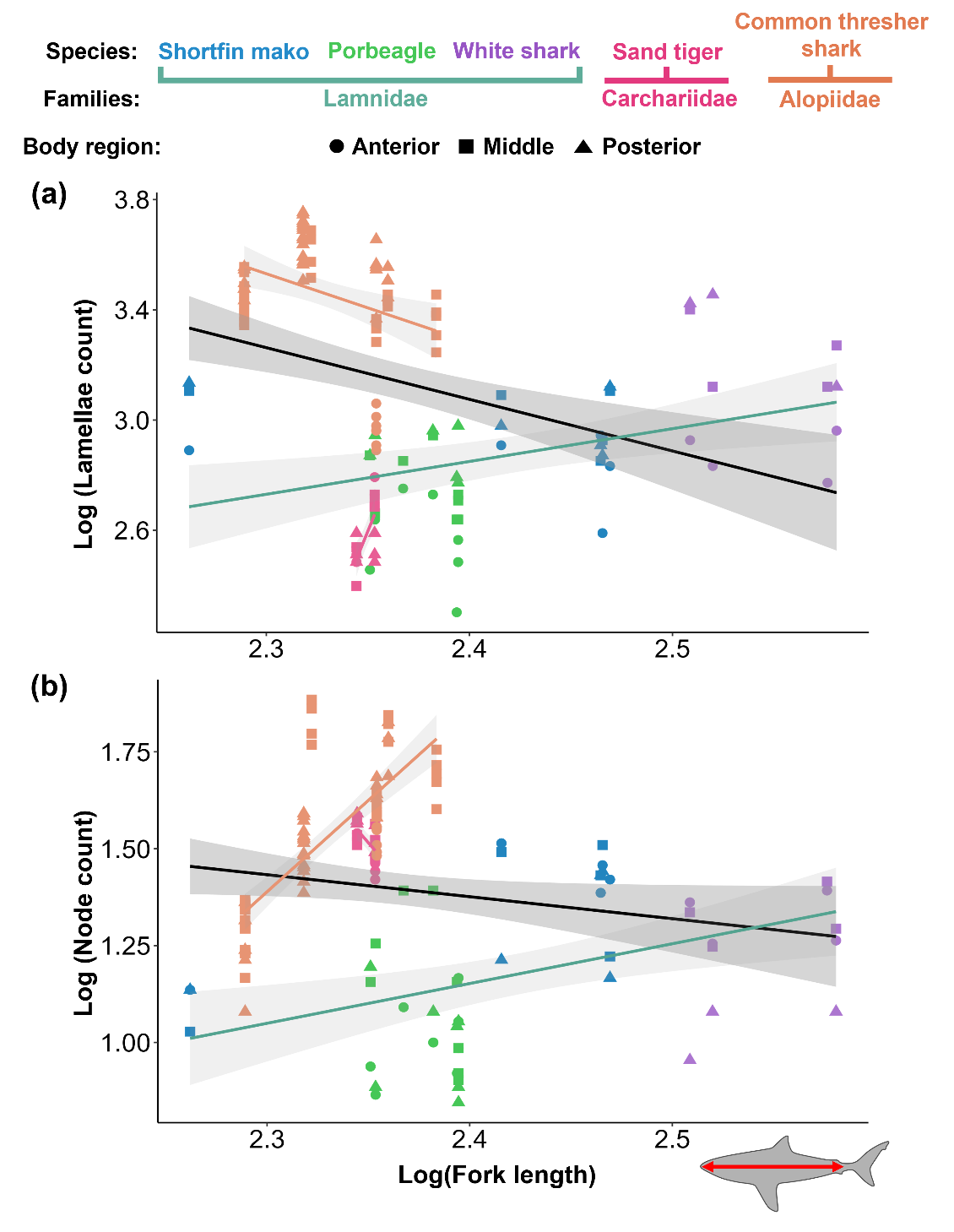


**Supplementary Figure 5:** Log-transformed linear regressions for fork length and (a) lamellae count, and (b) node count. Regression models for all species (black line) were significant for (a) lamellae (*F*_1,135_ = 16.010; *p*<0.001), but not for (b) nodes. Longer sharks are predicted to have less lamellae (*y* = -1.874*x* + 7.572; *R^2^* = 0.099). Point shape depicts body region and color represents species. Family-specific relationships are depicted by color regression lines (Alopiidae in orange, Carchariidae in pink, and Lamnidae in teal). The shaded gray ribbon for each line represents the 95% confidence interval.
